# Supplementary material for: Deterministic Progenitor Behavior and Unitary Production of Neurons in the Neocortex
Source: Cell. 2014 Nov 6;159(4):775–88. doi: 10.1016/j.cell.2014.10.027 (PMC4225456; doi:10.1016/j.cell.2014.10.027)
Supplement: Document S1. Article plus Supplemental Information [file mmc5.pdf]

Article

Cell

Deterministic Progenitor Behavior and Unitary Production of Neurons in the Neocortex

Peng Gao,[1,2,10] Maria Pia Postiglione,[3,10] Teresa G. Krieger,[4,5] Luisirene Hernandez,[1,2] Chao Wang,[6] Zhi Han,[7] Carmen Streicher,[3] Ekaterina Papusheva,[3] Ryan Insolera,[1,2] Kritika Chugh,[1] Oren Kodish,[1] Kun Huang,[6] Benjamin D. Simons,[4,5,8,11] Liqun Luo,[9,11] Simon Hippenmeyer,[3,11,*] and Song-Hai Shi[1,2,11,*]

[1]Developmental Biology Program, Memorial Sloan Kettering Cancer Center, 1275 York Avenue, New York, NY 10065, USA
[2]Graduate Program in Neuroscience, Weill Cornell Medical College, 1300 York Avenue, New York, NY 10065, USA
[3]Institute of Science and Technology Austria, Am Campus 1, 3400 Klosterneuburg, Austria
[4]Cavendish Laboratory, Department of Physics, J.J. Thomson Avenue, University of Cambridge, Cambridge CB3 0HE, UK
[5]Wellcome Trust/Cancer Research UK Gurdon Institute, University of Cambridge, Tennis Court Road, Cambridge CB2 1QN, UK
[6]Departments of Biomedical Informatics and Electrical and Computer Engineering, The Ohio State University, Columbus, OH 43210, USA
[7]College of Software, Nankai University, 94 Weijin Road, Tianjin 300071, P.R.C.
[8]Wellcome Trust-Medical Research Council Cambridge Stem Cell Institute, University of Cambridge, Cambridge CB2 1QR, UK
[9]Howard Hughes Medical Institute and Department of Biology, Stanford University, Stanford, CA 94305, USA
[10]Co-first author
[11]Co-senior author
*Correspondence: simon.hippenmeyer@ist.ac.at (S.H.), shis@mskcc.org (S.-H.S.)
http://dx.doi.org/10.1016/j.cell.2014.10.027
This is an open access article under the CC BY license (http://creativecommons.org/licenses/by/3.0/).

SUMMARY

Radial glial progenitors (RGPs) are responsible for producing nearly all neocortical neurons. To gain insight into the patterns of RGP division and neuron production, we quantitatively analyzed excitatory neuron genesis in the mouse neocortex using Mosaic Analysis with Double Markers, which provides single-cell resolution of progenitor division patterns and potential in vivo. We found that RGPs progress through a coherent program in which their proliferative potential diminishes in a predictable manner. Upon entry into the neurogenic phase, individual RGPs produce ∼8–9 neurons distributed in both deep and superficial layers, indicating a unitary output in neuronal production. Removal of OTX1, a transcription factor transiently expressed in RGPs, results in both deep- and superficial-layer neuron loss and a reduction in neuronal unit size. Moreover, ∼1/6 of neurogenic RGPs proceed to produce glia. These results suggest that progenitor behavior and histogenesis in the mammalian neocortex conform to a remarkably orderly and deterministic program.

INTRODUCTION

The mammalian neocortex commands all higher-order brain functions. It consists of an extraordinarily large number of excitatory and inhibitory neurons organized into distinct laminae. Previous studies showed that radial glia in the ventricular zone (VZ) of the developing neocortex are the progenitors that produce nearly all excitatory neurons (Kriegstein and Alvarez-Buylla, 2009). Prior to neurogenesis, radial glial progenitors (RGPs) divide symmetrically to amplify the progenitor pool. During the neurogenic phase, RGPs are believed to divide asymmetrically to produce neurons either directly or indirectly through transient amplifying progenitors, such as intermediate progenitors (IPs) (Florio and Huttner, 2014). Consecutive waves of neurogenesis lead to the formation of cortical layers in an "inside-out" fashion; that is, late-born neurons migrate past early-born neurons and progressively occupy more superficial layers (Angevine and Sidman, 1961). Although these studies have outlined a framework for our understanding of neocortical neurogenesis, precise knowledge of neuron production and organization, especially at the single-progenitor level, remains elusive.

Proper functioning of the neocortex depends on the production and positioning of the correct number and diversity of neurons for intricate circuit assembly. To generate a neocortex of the appropriate size and cellular composition, an exquisite balance must be reached between the proliferation and differentiation of RGPs. This balance could be regulated at the level of individual RGPs, which might undergo defined sequences of fate choices during progenitor amplification and neurogenesis. However, recent studies in adult mammalian tissues, including the epidermis (Clayton et al., 2007), airway epithelium (Teixeira et al., 2013), germline (Klein et al., 2010), and intestine (Snippert et al., 2010), suggest that a balance between proliferation and differentiation can also be achieved at the level of the stem/progenitor cell population. In this case, the behavior of individual progenitors appears to be stochastic, whereas the dynamics of the total population unfolds in a predictable manner.

Interestingly, a similar scenario has been proposed in the developing zebrafish retina (He et al., 2012).

Excitatory neurons in the neocortex are diverse in their dendrite morphology, axonal projection, and biophysical properties. This diversity is strongly tied to the histogenesis of the neocortex (Greig et al., 2013; Kwan et al., 2012). Early-born neurons, occupying the deep layers (5–6), are predominantly composed of corticofugal neurons that project away from the neocortex to subcortical targets, such as thalamus, brainstem, and spinal cord. On the other hand, late-born neurons, occupying the superficial layers (2–4), are largely composed of intracortical neurons that project locally or to the contralateral cortical hemisphere. The overall coupling between histogenesis and neuronal subtypes implies that RGPs, as a population, progress through a succession of states, and the probabilities of generating distinct neuronal types change as a function of time and/or cell division. This "progressive competence restriction" model was supported by previous progenitor transplantation studies (Desai and McConnell, 2000; Frantz and McConnell, 1996). In addition, dissociated and embryonic stem cell-derived cortical progenitors cultured in vitro recapitulate the sequential production of neuronal types as observed in vivo (Eiraku et al., 2008; Gaspard et al., 2008; Shen et al., 2006).

A number of neuronal type-specific transcription factors are already expressed in progenitors during early neocortical development (Greig et al., 2013; Kwan et al., 2012), raising the possibility that distinct subpopulations of progenitors are responsible for producing particular types of neocortical excitatory neurons. For example, orthodenticle homolog 1 (OTX1), a homeodomain transcription factor, is selectively expressed in a subset of subcerebral neurons in layer 5, as well as a number of neurons in layer 6, and regulates their axonal projection (Frantz et al., 1994; Weimann et al., 1999). Interestingly, OTX1 is also abundantly expressed in the VZ progenitors during the period of deep-layer neuron production, and its expression in progenitors is greatly reduced during the generation of superficial-layer neurons (Frantz et al., 1994). On the other hand, the POU (Pit-Oct-Unc)-homeodomain transcription factors POU3F3/BRN1 and POU3F2/BRN2, markers largely specific for superficial-layer neurons, are expressed in VZ progenitors during superficial-layer neurogenesis and regulate the specification and migration of superficial-layer neurons (Dominguez et al., 2013). Recent genetic fate-mapping experiments have suggested that, on the population level, progenitors expressing cut-like homeobox 2 (CUX2), another marker specific for callosal and other superficial-layer neurons, exclusively produce superficial-layer neurons (Franco et al., 2012), although conflicting results were subsequently reported (Guo et al., 2013). Therefore, further investigation using additional and independent genetic lineage tracing approaches, especially at the single progenitor level, is necessary to faithfully uncover the precise lineage relationship of neocortical progenitors and neurons. Besides neurons, RGPs also produce glial cells, including astrocytes and oligodendrocytes (Anthony et al., 2004; Kessaris et al., 2006), which have diverse roles in the development and maintenance of neurological function. Although gliogenesis is generally known to follow neurogenesis in the developing mammalian brain (Costa et al., 2009; Rowitch and Kriegstein, 2010), their precise relationship in vivo at the individual progenitor level remains largely unexplored.

Clonal analysis, through labeling of individual progenitor cells and following their progeny in the developing neocortex in vivo, could provide definitive answers to the ontogeny of neocortical neurons and glia. Indeed, previous clonal studies using retroviral labeling or chimeric mice have contributed to the current framework of neocortical neurogenesis (Kornack and Rakic, 1995; Luskin et al., 1988; McCarthy et al., 2001; Price and Thurlow, 1988; Tan et al., 1998; Walsh and Cepko, 1988, 1992). However, the lack of cellular resolution of progeny cell fate, vital for dissecting progenitor division patterns, and the imprecise spatiotemporal control of clonal labeling have so far precluded a definitive understanding of this complex and dynamic process. The MADM (*M*osaic *A*nalysis with *D*ouble *M*arkers) technique offers a solution (Hippenmeyer et al., 2010; Zong et al., 2005). In this study, we exploited the unprecedented resolution of MADM labeling and performed a quantitative clonal analysis of RGP division and lineage progression in the mouse neocortex.

RESULTS

MADM Analysis of Neocortical Neurogenesis

In MADM, Cre recombinase-mediated interchromosomal recombination in the G_2 phase of dividing progenitors followed by X-segregation (G_2-X, segregation of recombinant sister chromatids into separate daughter cells) reconstitutes one of two fluorescent markers, enhanced GFP (EGFP, green) or tandem dimer Tomato (tdTomato, red), in each of the two daughter cells (Figure S1A available online) (Zong et al., 2005). As such, G_2-X MADM events result in permanent and distinct labeling of the two descendent lineages, thereby allowing a direct assessment of the division pattern (symmetric versus asymmetric) and potential (the number of progeny) of the original dividing progenitors. In addition, upon G_2-Z (congregation of recombinant sister chromatids into the same daughter cell), G_1, or G_0 recombination events, green and red fluorescent proteins are restored simultaneously in the same cell, resulting in double-labeled (yellow) cells (Figure S1A).

To specifically label neocortical excitatory neuron progenitors in a temporally controlled manner, we introduced the *Emx1-CreER*^{T2} transgene (Kessaris et al., 2006) into the MADM system and induced Cre activity through a single dose of tamoxifen (TM) administered to timed pregnant females at one of the following four embryonic stages: E10, E11, E12, and E13 (Figure S1B). Brains were analyzed at either postnatal day (P)7–P10 or P21–P30. We found no labeling in the absence of TM treatment (n = 5 brains). To ensure unequivocal clonal analysis, we titrated the TM dose to achieve very sparse labeling (see below). To recover all labeled cells, we performed serial sectioning and three-dimensional (3D) reconstruction of individual brains (Figure 1A).

As expected, cells fluorescently labeled in green, red, or yellow exhibited the characteristic morphological features of neocortical excitatory neurons (Figures 1B and S2A). For example, in the entire hemisphere of a P21 brain exposed to TM at E10 (E10–P21), we observed only two clusters of neurons

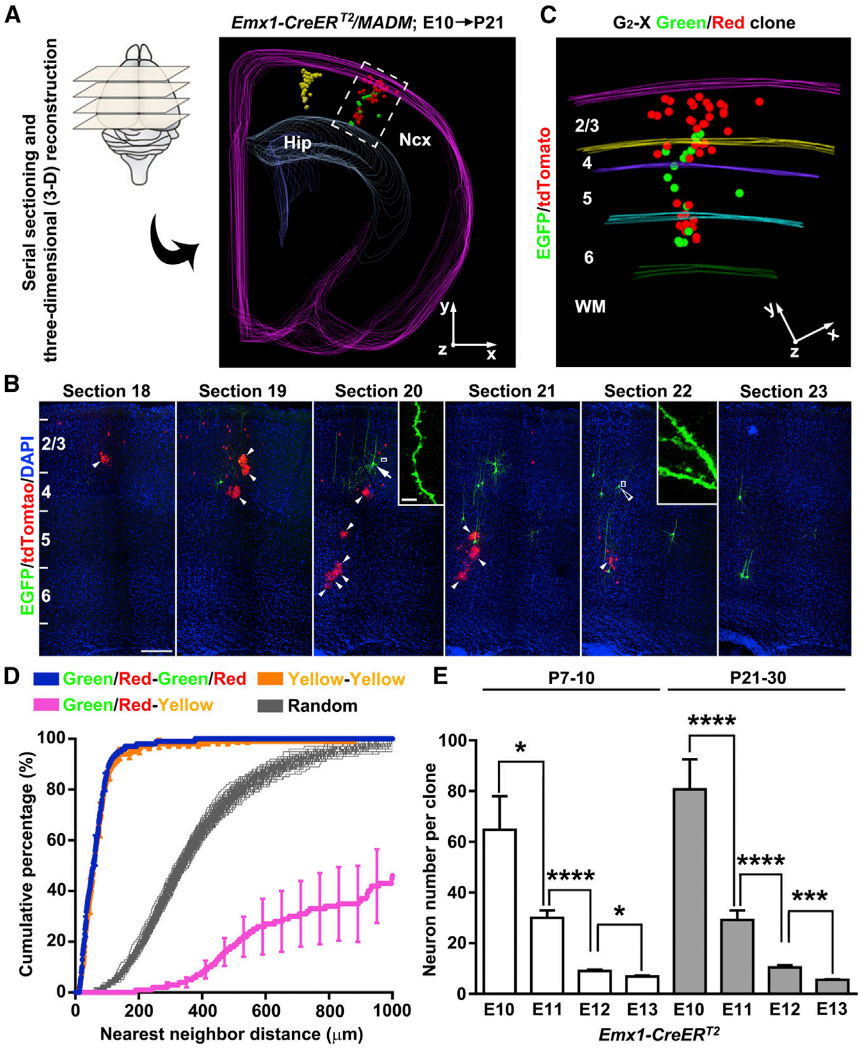

Figure 1. Clonal Analysis of Neocortical Excitatory Neuron Genesis and Organization Using MADM

(A) Serial sectioning and 3D reconstruction of a MADM-labeled P21 brain treated with TM at E10. Colored lines indicate the contours of the brain and colored dots represent the cell bodies of labeled neurons. The x/y/z axes indicate the spatial orientation of the clone with the y axis parallel to the brain midline and pointing dorsally. Similar display is used in subsequent 3D reconstruction images. Hip, hippocampus; Ncx, neocortex.

(B) Confocal images of the green/red G_2-X clone. Consecutive brain sections were stained with the antibodies against EGFP (green) and tdTomato (red) and with DAPI (blue). Layers are shown to the left. Arrow indicates an excitatory pyramidal neuron with a prominent apical dendrite, and open arrowhead indicates an excitatory stellate neuron. Arrowheads indicate glial cells. High-magnification images of their dendrites with numerous spines are shown in insets. Scale bars, 200 μm and 10 μm.

(C) High-magnification 3D reconstruction image of the green/red G_2-X clone. Colored lines indicate the layer boundary. WM, white matter.

(D) NND analysis of MADM-labeled neurons in the P21-30 neocortex treated with TM at E10. Data are presented as mean ± SEM.

(E) Quantification of MADM clone size (P7–P10: E10, n = 24; E11, n = 69; E12, n = 48; E13, n = 28; P21–P30: E10, n = 22; E11, n = 38; E12, n = 47; E13, n = 25).

Data are presented as mean ± SEM. (*$p < 0.05$, ***$p < 0.001$, and ****$p < 0.0001$). See also Figures S1 and S2 and Movies S1 and S2.

separated by 2,170 μm (Figure 1A and Movie S1), one consisting of green and red neurons (Figure 1C and Movie S2) and the other consisting of yellow neurons (Figures S2A and S2B). There were no scattered neurons or mixed clusters of green or red neurons with yellow neurons. Similar labeling efficiency and distribution patterns were found in another five brains treated with TM at E10 and analyzed at P21–30. In total, from these brains, we recovered 33 green/red fluorescent neuron clusters, 10 yellow fluorescent neuron clusters, and no mixed clusters with both green/red and yellow fluorescent neurons. To further quantitatively assess the spatial distribution and clonal relationship of labeled neurons, we applied nearest-neighbor distance (NND) analysis to the 3D reconstruction data sets (Brown et al., 2011; Diggle, 2003). The NNDs among the green/red neurons (blue) or the yellow neurons (yellow) were significantly shorter than that of simulated random data sets (Figure 1D). In contrast, the NNDs among the green/red and yellow neurons (magenta) were substantially longer. Together, these results demonstrated that clonally related neurons originating from distinct, sparsely labeled progenitors form spatially segregated clusters, and each cluster represents a clone that arises from a single neural progenitor. Notably, glial cells were also observed in some clones (arrowheads, Figures 1B and S2A).

We focused our analysis on green/red G_2-X clusters of clonally related neurons, as they provide crucial information on the division pattern and lineage potential of labeled progenitors, which would be otherwise unavailable from a conventional labeling strategy. We quantified the size of clones labeled at different embryonic stages and found that the average clone size decreased progressively as development proceeded (Figure 1E), which is consistent with an overall reduction in the proliferative and neurogenic potential of progenitors over time. We occasionally observed G_2-X clones containing only green or red neurons (Figure S3A), likely due to the apoptosis of one of the original two daughter cells. The overall rate of apoptosis in the neocortical excitatory neuron lineage appeared to be low (Figures S3B–S3E).

Unitary Production of Excitatory Neurons from RGPs

Previous work has suggested that, during early neocortical development, RGPs divide either symmetrically to amplify their number or asymmetrically to produce neurons while self-renewing (Florio and Huttner, 2014). MADM clonal analysis validated

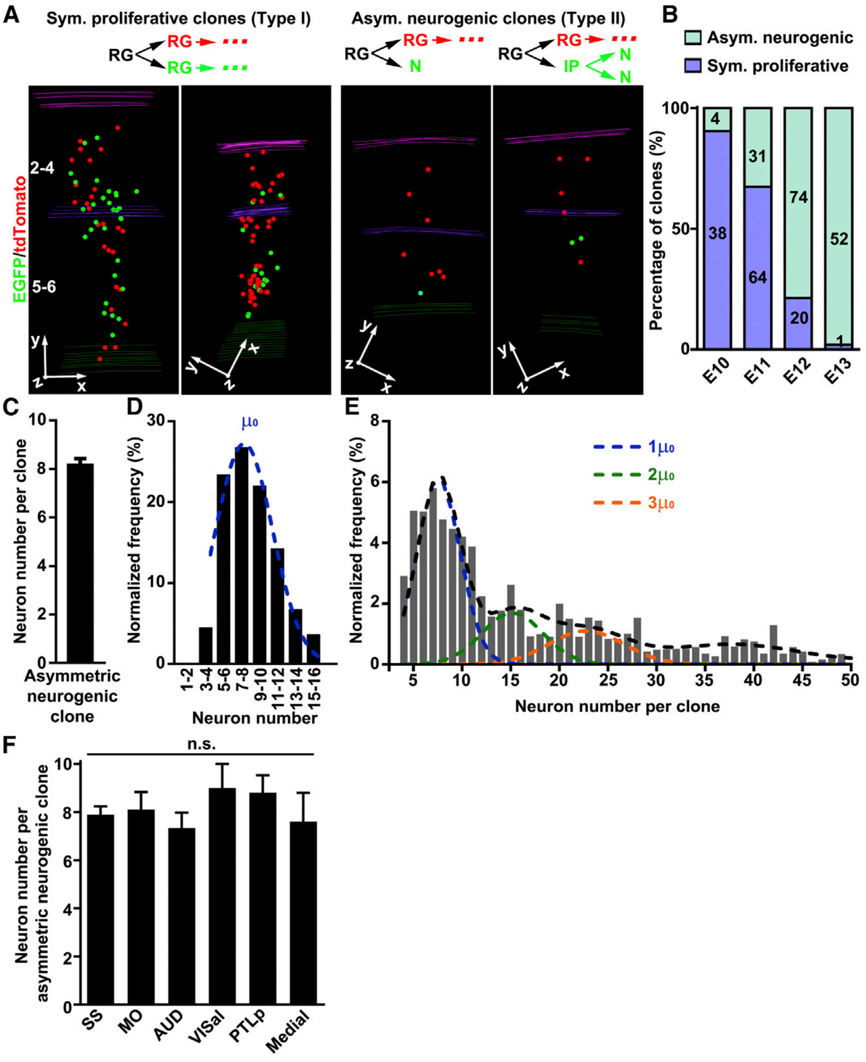

Figure 2. Unitary Production of Excitatory Neurons by RGPs

(A) 3D reconstruction images of representative symmetric proliferative (left) and asymmetric neurogenic (right) clones. Schematics of the clone are shown at the top. RG, radial glia; N, neuron; IP, intermediate progenitor.

(B) Percentage of symmetric proliferative division versus asymmetric neurogenic division at different embryonic stages.

(C) Quantification of the size of asymmetric neurogenic clones labeled at E10–E12 (n = 109).

(D) Clone size distribution of the asymmetric neurogenic clones at E10–E12 fitted by a Gaussian distribution, indicating an average RGP output of ~8–9 neurons (mean μ_0 = 8.4, SD δ = 2.6; fitting error = 5.3%; blue broken line; termed "Unitary Gaussian").

(E) Gaussian fitting of the overall clone size variation. The 192 clones with a size of up to 50 neurons were fitted by the sum (black line) of a series of Gaussians centered on integer multiples of the mean of Unitary Gaussian in D ($1\mu_0$, $2\mu_0$, $3\mu_0$; colored lines; higher-order Gaussians are not plotted for clarity).

(F) Quantification of the size of asymmetric neurogenic clones located in different neocortical areas (SS, 7.9 ± 0.3, n = 44; MO, 8.1 ± 0.7, n = 10; AUD, 7.3 ± 0.6, n = 15; VISal, 9.0 ± 1.0, n = 2; PTLp, 8.8 ± 0.7, n = 5; Medial, 7.6 ± 1.2, n = 10). SS, somatosensory cortex; MO, motor cortex; AUD, auditory cortex; VISal, visual cortex; PTLp, posterior parietal association areas; Medial, including anterior cingulate area, dorsal peduncular area, infralimbic area, prelimbic area, and retrosplenial area.

Data are presented as mean ± SEM. n.s., not significant. See also Figures S3 and S4.

these observations. We found that G_2-X clones could be grouped into two types. They either contained sizable numbers of both green and red neurons (more than three neurons of each color) distributed throughout the superficial and deep layers (termed type I, Figure 2A, left) or else contained a "majority" population in one color and a "minority" population (less than four neurons, mostly one or two neurons) in the other color (termed type II, Figure 2A, right). Interestingly, in type II clones, the minority population always resided in the deeper layers relative to the majority population. Consistent with the "inside-out" sequence of neocortical neurogenesis (Angevine and Sidman, 1961), the minority population thus represents the earliest-born neurons in the labeled lineage. Moreover, the relative scarcity of the minority population indicates that the original daughter cell, from which the minority population arises, is either a neuron or an IP capable of undergoing only one to two rounds of division (i.e., producing no more than four neuronal progeny) (Noctor et al., 2004), whereas the majority population originates from a self-renewing RGP. Therefore, type II clones represent asymmetric neurogenic clones (Figure 2A, right, top). In contrast, type I clones represent symmetric proliferative clones, as the two originally labeled daughter cells are most probably self-renewing RGPs, each capable of producing a large cohort of neuronal progeny (Figure 2A, left, top).

The exquisite resolution of G_2-X clones allowed a direct quantitative measurement of symmetric proliferative and asymmetric neurogenic division frequencies of RGPs in vivo. We found that the transition from symmetric proliferation to asymmetric neurogenesis occurs predominantly at E11–E12 (Figure 2B). Importantly, the explicit identification of asymmetric neurogenic clones (type II) enabled a quantitative assessment of the neurogenic capacity of individual RGPs as they switch from symmetric proliferative division to asymmetric neurogenic division. We found that the average size of asymmetric neurogenic clones labeled by TM treatment at E10–E12 was ~8–9 neurons (Figure 2C). Moreover, the histogram of individual asymmetric neurogenic clone sizes could be described by a Gaussian-like distribution (which we term "Unitary Gaussian"; centered on a "mean" value of [μ_0 =]8.4 neurons and a SD of [δ =]2.6 neurons; Figure 2D). The appearance of a defined peak at eight to nine neurons

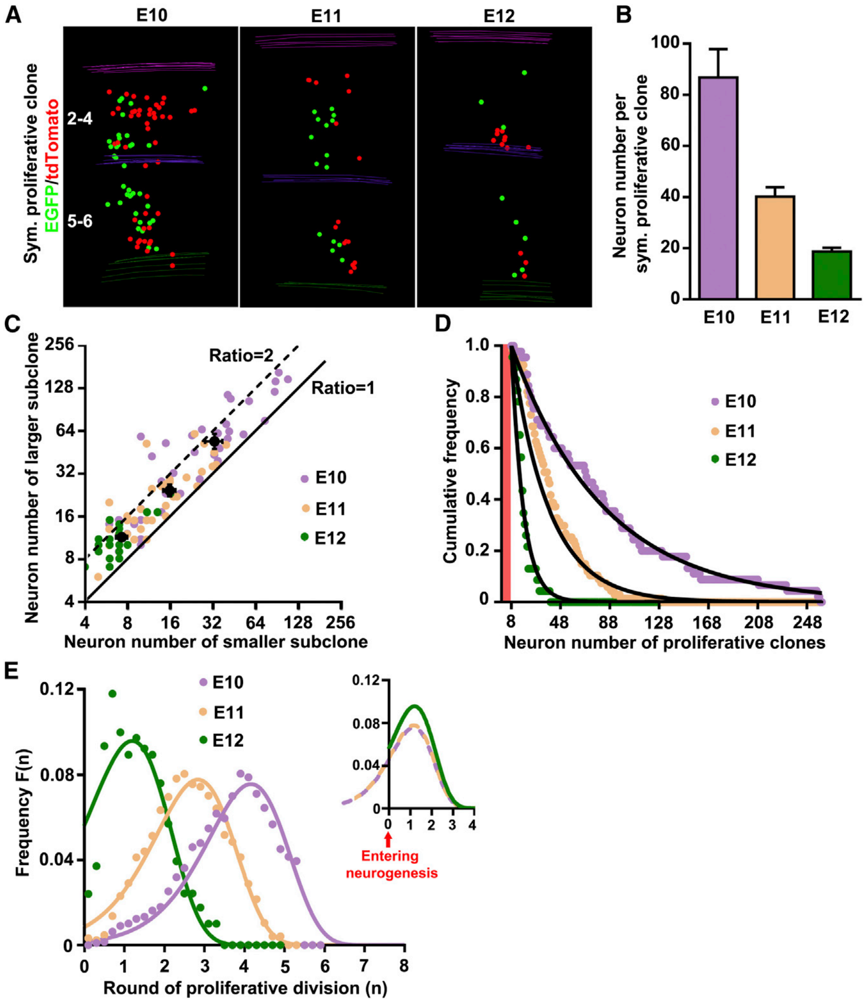

Figure 3. Defined Temporal Program in Diminishing Proliferative Potential by RGPs

(A) 3D reconstruction images of representative type I symmetric proliferative clones labeled at different developmental stages.

(B) Quantification of the size of symmetric proliferative clones labeled at E10 (n = 38), E11 (n = 64), and E12 (n = 20). Data are presented as mean ± SEM.

(C) Scatterplot of the size of the larger versus smaller sister subclones of individual symmetric proliferative clones. Black dots and bars represent the mean and SEM at each developmental stage.

(D) Cumulative frequency distribution of the size of symmetric proliferative clones labeled at different developmental stages. Red shaded area indicates no proliferative clone with a size less than eight neurons.

(E) Normalized distribution of the round(s) of symmetric proliferative division of the founder RGPs in symmetric proliferative clones prior to neurogenic division. Dots represent individual proliferative clones, and lines represent the estimated distribution of the clones based on the exponential fitting in (D). The overlay of lines is shown in the inset (n = 0 indicates entering neurogenesis).

contrasted with what one would expect if RGPs in the neurogenic phase stochastically undergo terminal differentiation (i.e., exit cell cycle) at any time in a generation-independent manner, as this would result in a geometric (i.e., negative exponential) distribution of clone sizes with a peak at the smallest clone size. Instead, our data suggest that individual RGPs do not exit the cell cycle randomly but have a defined unitary output in neuronal production after they enter the neurogenic phase.

Should this be the case, one would predict that symmetric proliferative divisions of RGPs prior to asymmetric neurogenic divisions predominantly produce clones with a size that is an integer multiple of the Gaussian unit. Indeed, we found that the overall size distribution for a majority of clones labeled at E10–E12 could be described as a series of Gaussians centered on integer multiples of the Unitary Gaussian mean (Figure 2E, black and colored lines), suggesting that excitatory neurons in the neocortex are produced in "quanta" (i.e., around eight to nine neurons) by RGPs. Interestingly, the size of asymmetric neurogenic clones was similar across different neocortical areas (Figure 2F), suggesting that the unitary neuronal output is a general property of RGPs.

Similar results in excitatory neuron clonal labeling and properties were obtained when we used *Nestin-CreER*^{T2} (Figure S4), another transgenic mouse line with neural progenitor-specific expression of TM-inducible Cre (Imayoshi et al., 2006). Notably, we occasionally observed labeled neocortical interneurons in *Nestin-CreER*^{T2}*;MADM* brains that were readily distinguished based on morphology.

Coherent and Predictable Proliferation Program by RGPs

Turning to the quantitative analysis of the behavior of type I symmetric proliferative clones (Figure 3A), we found that their average size progressively decreased over time (Figure 3B). Notably, two self-renewing RGPs produced by a "symmetric" proliferative division can have distinct proliferative and neurogenic potentials, as reflected by the differences in the number and spatial distribution of their neuronal progeny (green versus red subclones; Figure 3A). Remarkably, however, the sizes of sister subclones lay mostly within a factor of two from each other, i.e., less than one round of cell division apart (Figure 3C). Moreover, the mean ratio of the larger to smaller sister subclone size was surprisingly similar regardless of the total clone size or the time of induction (E10, 1.7 ± 0.2; E11, 1.6 ± 0.1; E12, 1.6 ± 0.1; p = 0.6; Figures 3C, black dots), suggesting that the proliferative potential of sister RGPs is similarly correlated at all stages. If RGPs transitioned into the neurogenic phase in a sporadic stochastic manner, one would expect the size of sister subclones to

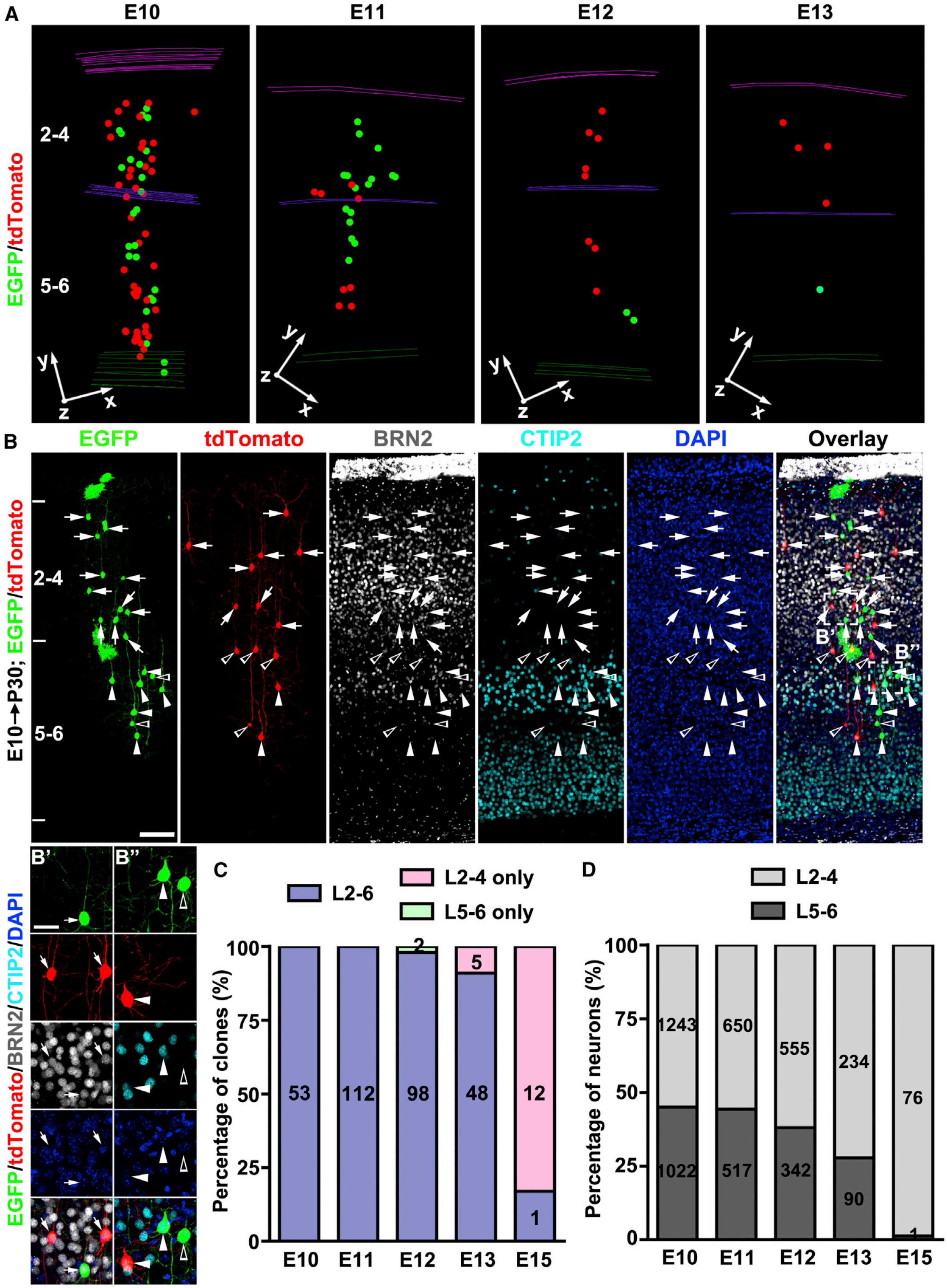

Figure 4. Individual RGPs Produce Both Deep- and Superficial-Layer Excitatory Neurons

(A) 3D reconstruction images of representative clones labeled at different embryonic stages. Note that all clones contain both superficial (2–4) and deep (5–6) layer neurons.

(B) Confocal images of an E10 clone stained with the antibodies against EGFP (green), tdTomato (red), BRN2 (white), and CTIP2 (cyan) and with DAPI (blue). High-magnification images of representative superficial (B') and deep (B'') layer neurons (broken lines) are shown at the bottom. Arrows indicate neurons

(legend continued on next page)

become decorrelated. Consistently, random pairing of subclones at each developmental stage led to drastically different ratios of larger to smaller subclone size. Thus, these results strongly suggest that the correlation between sister subclone sizes does not occur randomly but instead indicate that the proliferative potential of RGPs diminishes coherently and synchronously across generations.

To search for further evidence of a coherent underlying program in the proliferative phase, we analyzed the size distribution of symmetric proliferative clones. Interestingly, the cumulative distribution of clone sizes could be well described by an exponential distribution at each time point (Figure 3D). Based on the unitary neuronal output (Figure 2), we estimated the rounds of proliferative division that RGPs underwent prior to neurogenic division to produce the clone (see Experimental Procedures). Importantly, we found that the corresponding distribution of proliferative divisions of individual RGPs at each time point was peaked with a width of ~2.5 cell divisions (Figure 3E), indicating only a moderate degree of variation in proliferative capacity among RGPs induced at the same time. Moreover, the shape of the distribution was nearly identical at each time point, with the peak position shifting over time in a predictable manner (Figure 3E). Based on this distribution, we estimated that RGPs induced at E10, E11, and E12 proliferated on average for ~3.8, ~2.6, and ~1.3 rounds of division, respectively, before entering into the neurogenic phase. These results provide direct and quantitative in vivo evidence that the proliferative capacity of RGPs diminishes progressively in a defined, coherent program over time.

Laminar Distribution of Clones

A recent study suggested that RGPs are fate restricted to selectively produce only superficial- or deep-layer neurons (Franco et al., 2012). We found that, regardless of the time of labeling and clone size, the vast majority of clones spanned both the deep (5–6) and superficial (2–4) layers (Figure 4A). Besides the assessment of spatial localization, we also performed immunohistochemical analysis using antibodies against well-established layer-specific neuronal markers (Greig et al., 2013; Kwan et al., 2012). BRN2 or CUX1 (superficial layer marker)- and CTIP2 (deep layer marker) -positive neurons were found to coexist in individual clones (Figures 4B and S5). We also observed subplate neurons (SPNs) in clones labeled at E10–E11, which were grouped with other deep-layer neurons (Figures S2C and S2D).

We quantified the percentage of clones that contained both superficial and deep-layer neurons, as well as clones with neurons only in deep or superficial layers (Figure 4C). All clones labeled at E10 and E11 contained both superficial and deep layer neurons. About 98% and 91% of clones labeled at E12 and E13 contained both superficial and deep-layer neurons, respectively. When induced at E15, 12 out of 13 clones contained only superficial layer neurons (Figure 4C). Consistent with the "inside-out" sequence of neocortical neurogenesis, we observed a gradual shift of labeled neurons in the clones from deep layers to superficial layers (Figure 4D).

OTX1 Regulates Neuronal Unit Size

The MADM technique also enables mosaic knockout studies for genes located on the same chromosome as the MADM cassettes (Zong et al., 2005). By genetically linking one fluorescent marker to the wild-type allele and the other fluorescent marker to the knockout allele, the cell-autonomous function of a gene of interest can be assessed. To gain more insight into the molecular regulation of RGP behavior and unitary neuronal production, we examined the function of OTX1, a transcription factor selectively expressed in RGPs during the period of deep-layer neuron production (Frantz et al., 1994), using MADM mosaic knockout analysis.

We introduced the knockout allele of *Otx1* (Acampora et al., 1996) into the *Emx1-CreER*^{T2}*/MADM* system by genetically linking it to the GT cassette through meiotic recombination; in parallel, the TG cassette was linked to the wild-type allele (Figure 5A). As a result, upon Cre-mediated interchromosomal recombination, the two daughter cells of a RGP undergoing asymmetric neurogenic division exhibited distinct genotypes: the green daughter cell inherited the wild-type allele, whereas the red daughter cell inherited the *Otx1* knockout allele. Depending on whether the red daughter cell was a renewing RGP or a differentiating daughter cell (neuron or IP), there were two possible clonal outcomes (Figure 5A).

To analyze the unitary neuronal output of RGPs in the absence of OTX1, we injected TM at E11 or E12, when RGPs transit from symmetric proliferative division to asymmetric neurogenic division (Figure 2B). As expected, we observed asymmetric neurogenic clones with a majority of green neurons and a minority of red neurons (Figure 5B, left) or with a majority of red neurons and a minority of green neurons (Figure 5B, right). Because the two colors corresponded to different genotypes, we compared the size of the majority or minority neuron population in individual clones with respect to their color (i.e., genotype). Interestingly, the red majority neuron number (originating from an *Otx1*$^{-/-}$ self-renewing RGP) was significantly reduced when compared to the green majority neuron number (originating from a wild-type self-renewing RGP) in individual clones (Figure 5C), suggesting a decreased neurogenic capacity in *Otx1*$^{-/-}$ RGPs. There was no obvious difference between the red and green minority neuron number (Figure 5D), indicating that the neurogenic capacity of IPs and the survival of neurons are not affected.

Given that *Otx1* is predominantly expressed in RGPs when deep-layer neurons are produced, we tested whether the reduction of neuronal production in *Otx1*$^{-/-}$ clones is selective for deep-layer neurons. To our surprise, the reduction applied to both superficial-layer (Figure 5E) and deep-layer (Figure 5F)

positive for BRN2, arrowheads indicate neurons positive for CTIP2, and open arrowheads indicate neurons negative for BRN2 or CTIP2. Scale bars, 100 μm and 25 μm.

(C) Percentage of clones containing both superficial and deep-layer neurons versus those containing only superficial- or deep-layer neurons.

(D) Percentage of neurons in the clones located in superficial or deep layers.

See also Figure S5.

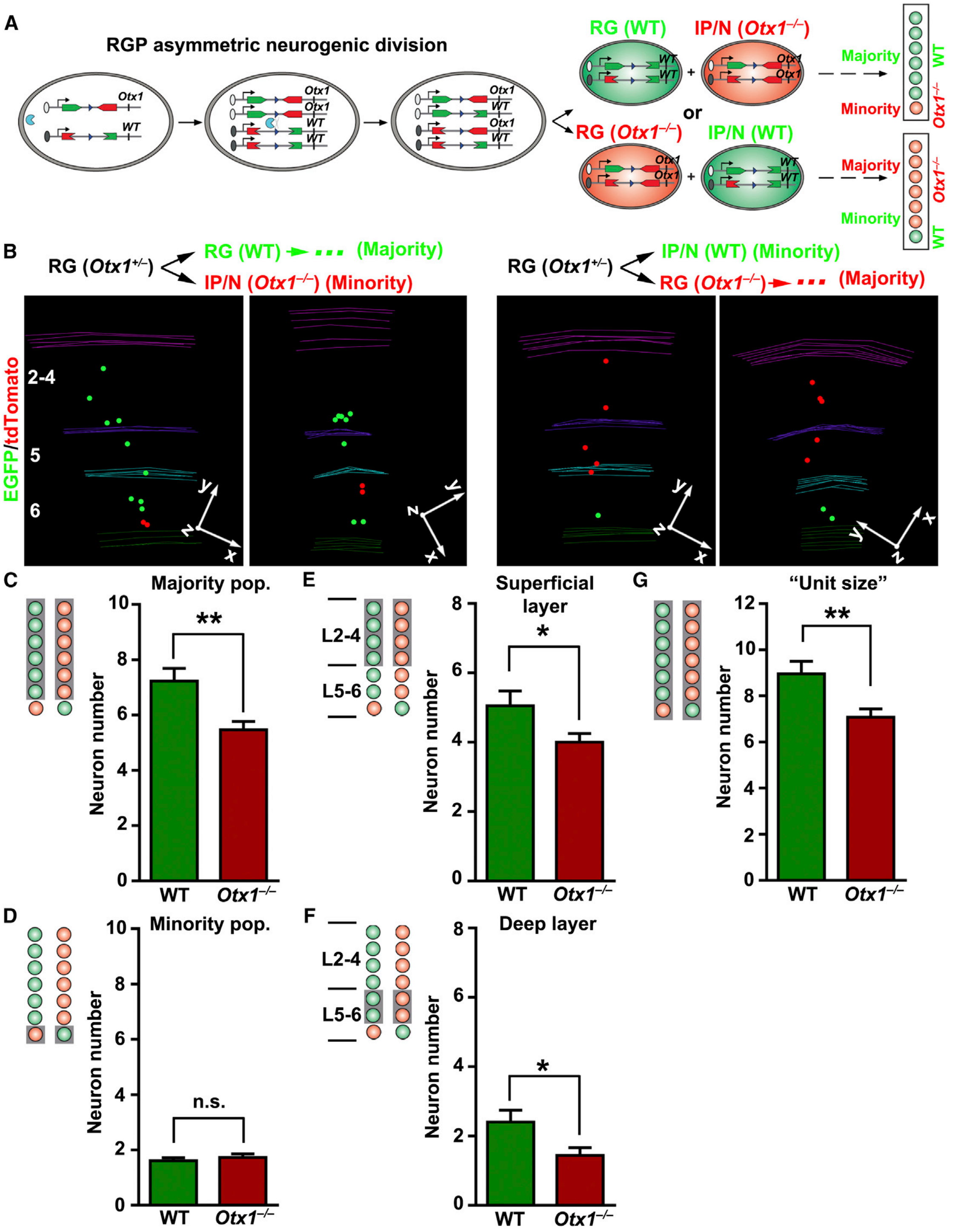

Figure 5. OTX1 Regulates the Production of Deep- and Superficial-Layer Neurons and Unitary Neuronal Output of RGPs

(A) Outline of MADM-based mosaic knockout analysis of *Otx1* in RGPs undergoing asymmetric neurogenic division. Note that tdTomato labels *Otx1*$^{-/-}$ cells and EGFP labels wild-type cells within the clone. RG, radial glia; N, neuron; IP, intermediate progenitor.

(B) 3D reconstruction images of representative G_2-X clones in mosaic *Otx1*-MADM neocortices. Schematics of the clone are shown at the top.

(C) Quantification of the size of the majority population arising from renewing RGPs in mosaic asymmetric neurogenic *Otx1*-MADM clones (**$p < 0.01$).

(D) Quantification of the size of the minority population arising from IPs or Ns in mosaic asymmetric neurogenic *Otx1*-MADM clones (n.s., not significant).

(legend continued on next page)

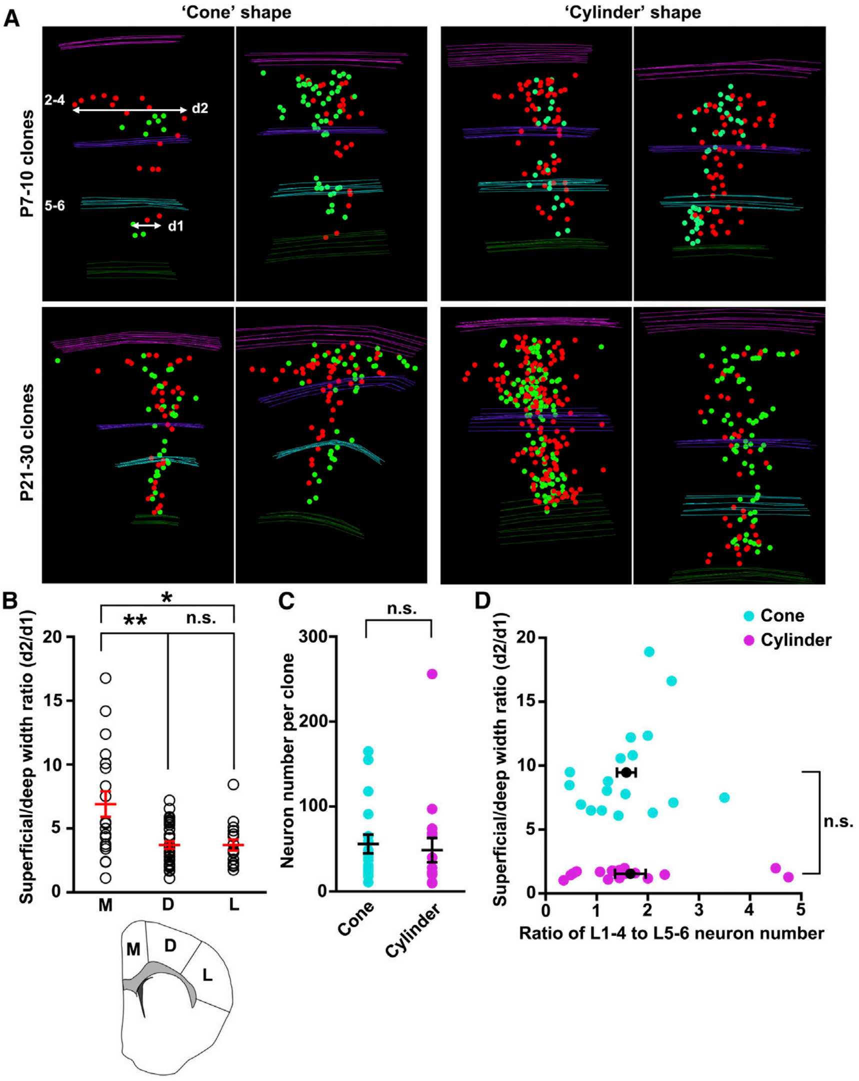

Figure 6. Spatial Organization of Neocortical Excitatory Neuron Clones

(A) 3D reconstruction images of representative clones that are "cone" shaped (left) and "cylinder" shaped (right) at P7–P10 (top) and P21–P30 (bottom) labeled at E10–E12.

(B) Quantification of the ratio of the maximal lateral dispersion in the superficial layer 2/3 in all dimensions (d2) to that in the deep layer 6 (d1) (see A) for clones located in different regions of the neocortex (medial [M], n = 19; dorsal [D], n = 33; lateral [L], n = 16; see inset at the bottom). Individual circles represent a single clone. Mean and SEM are shown in red (*p < 0.05 and **p < 0.01; n.s., not significant).

(C and D) No correlation between the clone shape and the clone size (C) or the ratio of neuron number in the superficial (2–4) and deep (5–6) layers (D). Each dot indicates a clone and black lines indicate mean ± SEM.

See also Movies S3 and S4.

neurons. Because removal of OTX1 did not affect the minority population, we considered the clones containing red neuron majority and green neuron minority as "*Otx1*$^{-/-}$ units," whereas those containing green neuron majority and red neuron minority were considered "wild-type units." Consequently, whereas the total size of "wild-type units" was similar to the unit size in wild-type animals (Figures 2C and 2D), the total size of "*Otx1*$^{-/-}$ units" was substantially decreased (Figure 5G). This decrease in unitary neuronal output by RGPs largely accounted for the microcephaly (Figures S6A and S6B) and reduced neocortical thickness (Figures S6C–S6F) observed in *Otx1*$^{-/-}$ animals (Acampora et al., 1996). Together, these results strongly suggest that OTX1 plays an important cell-autonomous role in controlling the production of both the deep and superficial layer neurons and the size of the unitary neuron output by individual RGPs.

Topological Organization of Clones

Although it has long been postulated that ontogenetic clonal units are the building blocks of the neocortex (Rakic, 1988), the precise topological organization of individual clonal units has not been determined. Our 3D reconstruction of individual clones permitted a quantitative analysis of the spatial organization of well-defined clones labeled at specific developmental stages and located in particular neocortical areas. Regardless of their size and location, all clones were organized into vertical clusters, which is consistent with a predominantly radial migration of neurons from their birthplace to the final destination (Figures 1C, 2A, 3A, and 4A). When we analyzed the clones labeled at E10–E12 that tended to be larger in size and thus offered more spatial features, we found that they exhibited distinguishable spatial organization patterns. Some clones were similarly dispersed laterally in deep and superficial layers (termed as "cylinder" shape), whereas other clones were substantially more dispersed in superficial layers than deep layers (termed as "cone" shape) (Figure 6A and Movies S3 and S4).

To reliably recapitulate the 3D organization of individual clones, we measured the ratio of the maximal lateral dispersion in the superficial layers 2/3 in all dimensions (d2) to that in the deep layer 6 (d1) for each clone (Figure 6A). Intriguingly, we noticed that clones located in the medial region, where the

(E) Quantification of the number of superficial-layer neurons in the majority population (*p < 0.05).

(F) Quantification of the number of deep-layer neurons in the majority population (*p < 0.05).

(G) Quantification of the unitary size of asymmetric neurogenic clones (*p < 0.05; n.s., not significant).

Data are presented as mean ± SEM in (C)–(G). (WT, n = 22 from 5 brains; *Otx1*$^{-/-}$, n = 28 from 5 brains). See also Figure S6.

neocortex bends, exhibited a higher ratio of d2/d1 than those in the dorsal and lateral regions (Figure 6B), indicating a potential link between clone shape and local geometry of the neocortex. It is important to note that clone shape did not seem to depend on the number (Figure 6C) or layer distribution of neurons (Figure 6D) in the clone. In spite of previous studies suggesting the tangential migration of clonally related excitatory neurons (Fishell et al., 1993; O'Rourke et al., 1992; Walsh and Cepko, 1992), we found them to be spatially packed into discrete vertical clusters with variable geometry, which likely play important roles in the structural and functional organization of the neocortex.

Clonal Relationship of Neurons and Glia

In addition to self-renewal and neuron production, RGPs also give rise to glial cells (Magavi et al., 2012; Qian et al., 2000; Rowitch and Kriegstein, 2010; Schmechel and Rakic, 1979). Consistent with this, we frequently found that spatially isolated clonal clusters induced at E10–E13 contained both neurons and glia, including astrocytes and oligodendrocytes (Figures 7A, 7B, and S7). Notably, although we observed clones with only neurons, we did not detect any clone with only glial cells, suggesting that glia-specific RGPs, if they exist, are extremely scarce and that gliogenesis consistently occurs after neurogenesis at the individual RGP level.

We next examined the relationship between neurogenesis and gliogenesis by comparing the neuronal and glial output of individual RGPs. Interestingly, the percentage of clones containing glia increased with the number of neurons in the clone (Figures 7C and S7), suggesting a coupling between the neurogenic capacity of an RGP and its gliogenic capacity. Moreover, the step-wise increase in gliogenic capacity fits well with our earlier observation of a unitary neuron output by individual RGPs. Clones with 4–12 neurons largely originated from ~1 neurogenic RGP, whereas clones with >49 neurons originated from ~6 neurogenic RGPs. Given that ~16% of clones with 4–12 neurons contained glia and ~96% of clones with >49 neurons contained glia, these results suggest that ~16% of individual neurogenic RGPs (i.e., ~1 in 6 RGPs) successfully proceeded to gliogenesis at the end of neurogenesis. Consistent with this, the fraction of asymmetric neurogenic clones that contained glia was ~16% (Figure 7D). Notably, in asymmetric neurogenic clones, the glia always shared the same color with the "majority" population of neurons in the clone, indicating that they arise from the asymmetrically dividing RGPs at the end of neurogenesis. Together, these results strongly suggest that a defined fraction (one in six) of neurogenic RGPs transition to gliogenesis upon the completion of neurogenesis, whereas the remaining RGPs exit the cell cycle through a terminal neurogenic division.

DISCUSSION

By using MADM-based quantitative clonal analysis of RGP lineage progression, we revealed a remarkably deterministic and coordinated developmental program of RGP behavior and neocortical histogenesis. These conclusions could only be inferred reliably by virtue of the lineage resolution provided by the MADM technique, as it allows the differential labeling of two original daughter cells and their progeny arising from individual dividing progenitors and thereby enables explicit in vivo assessment of RGP division pattern and potential. In conjunction with the CreER system, high-resolution clonal analysis can be performed in a temporally controlled and cell-type-specific manner. In this study, we used two CreER lines, *Emx1-CreER*^{T2} and *Nestin-CreER*^{T2}, both of which drive the broad expression of an inducible Cre enzyme in RGPs in the embryonic neocortex starting from ~E9–E9.5. We analyzed over 370 clones and obtained similar results for both CreER lines. Therefore, the features that we learned concerning RGP behavior and neurogenesis likely reflect the fundamental principles underlying neocortical excitatory neuron production and organization.

Our systematic in vivo clonal analysis suggests that the behavior of RGPs is remarkably coherent and predictable across all developmental stages. RGPs undergo symmetric proliferative division initially. At each embryonic time point (E10–E12), the proliferative capacity of RGPs peaked at a defined number of rounds of division, suggesting that the proliferative potential of individual RGPs at different embryonic stages can be reliably predicted. Moreover, the modest degree of variability in RGP proliferation that we observed at each induction time point remained virtually constant, indicating that the entire RGP population behaves similarly across different embryonic stages. Notably, at least part of the variability could derive from the TM processing and induction of MADM labeling. Thus, the intrinsic variability of RGPs could conceivably be less than experimentally estimated.

Our findings show that RGPs progressively and predictably lose their proliferative potential as development proceeds, as indicated by the decrease in the rounds of symmetric proliferative division from E10 to E11 and E12. This observation raises the intriguing possibility that the proliferative potential of RGPs may be controlled by a molecular clock that counts the time or the rounds of cell division, as suggested for neurogenesis of cerebellar granule cells (Espinosa and Luo, 2008). In support of this idea, we found that the proliferative potential of sister RGPs, although not exactly the same, was strongly correlated. Moreover, the degree of this correlation appeared to persist across different embryonic stages.

We found that RGPs transit from symmetric proliferative division to asymmetric neurogenic division around E11–E12. Once they enter the neurogenic phase, individual RGPs produce a defined number of neurons. Moreover, they produce deep-layer neurons before superficial-layer neurons. This unitary or quantal behavior of neuronal production by individual RGPs provides definitive evidence for the ontogeny of neocortical excitatory neurons, which has been much debated. Previous histological, transplantation, and in vitro progenitor and stem cell culture studies support a "progressive competence restriction" model involving the sequential production of neocortical neurons located in deep and superficial layers by RGPs (Desai and McConnell, 2000; Eiraku et al., 2008; Frantz and McConnell, 1996; Gaspard et al., 2008; Luskin et al., 1988; Qian et al., 1998; Shen et al., 2006). However, this model has been challenged by a recent genetic fate-mapping study (Franco et al., 2012) that argues for a "fate-restricted progenitor" model, proposing that RGPs are prespecified into distinct subpopulations

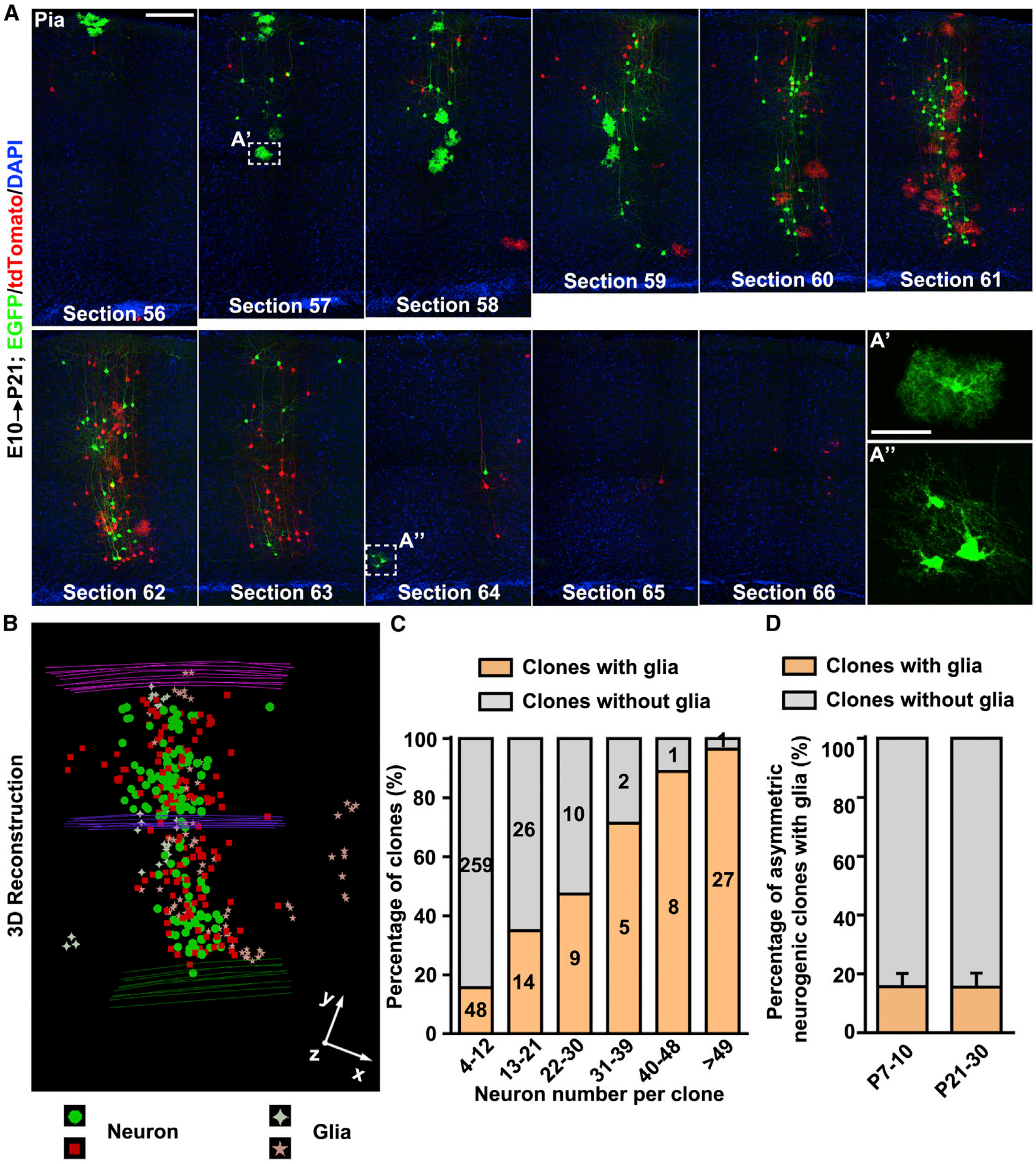

Figure 7. Predictable Rate of RGP Transitioning from Neurogenesis to Gliogenesis

(A) Confocal images of an E10–P21 green/red G_2-X clone that contains both green and red glial cells. High-magnification images of an astrocyte (A') and a few oligodendrocytes (A'') are shown in insets. Scale bars, 200 μm and 50 μm.

(B) 3D reconstruction image of the clone in (A).

(C) Percentage of all clones with or without glia with regard to the number of neurons in the clone.

(D) Percentage of asymmetric neurogenic clone with or without glia at P7–P10 and P21–P30.

Data are presented as mean ± SEM. See also Figure S7.

that are restricted to producing either superficial- or deep-layer neurons (Franco and Müller, 2013; Marín, 2012). Although we could not completely exclude the possibility of the existence of RGPs that produce only superficial- or deep-layer neurons, our extensive clonal analysis data clearly demonstrated the robust sequential production of deep- and superficial-layer neurons by individual RGPs.

To further address the neurogenic capacity of individual RGPs, we performed mosaic analysis at the single RGP level to selectively remove OTX1, a transcription factor predominantly expressed in RGPs at the early embryonic stage, as well as in deep-layer neurons postnatally (Frantz et al., 1994; Greig et al., 2013). Due to this unique expression pattern, OTX1 has been postulated to control the production of deep-layer neurons

specifically. To our surprise, removal of OTX1 led to a loss of both deep- and superficial-layer neurons and a reduction of the neuronal output unit size. The reduction in the unitary neuronal output of individual RGPs essentially accounted for the reduction in neocortical area and thickness by ~20%–30% observed in *Otx1*$^{-/-}$ mice. These results clearly suggest that OTX1 regulates the production of both deep- and superficial-layer neurons, despite its expression in RGPs being downregulated during superficial-layer neuron genesis. Our data thus provided a clear example that the early expression of a transcription factor in RGPs influences late neuronal production, which could be achieved by controlling the late expression of other critical factors required for the production of superficial-layer neurons. Importantly, these data suggest that the seemingly "correlated" expression of certain transcription factors in RGPs and postmitotic neurons does not necessarily reflect an exclusive lineage bias.

Finally, our results also demonstrate that, upon the completion of neurogenesis, individual RGPs proceed to gliogenesis at a predictable rate. This coupling between gliogenesis and neurogenesis may dictate the overall ratio of neurons to glia in the neocortex and thereby be critical for neocortical formation and function.

Our finding of a deterministic and linear progression of RGPs through proliferation, neurogenesis, and gliogenesis resonates with the temporal patterning of *Drosophila* neural progenitors/neuroblasts, where defined transcriptional regulation is known to control sequential production of diverse neural types (Bayraktar and Doe, 2013; Li et al., 2013). Future efforts toward unraveling additional molecular control of RGP division and lineage progression will be essential for understanding the programmed behavior of RGPs and the quantal nature of neocortical neurogenesis. Furthermore, recent studies demonstrated that the evolutionary expansion of the neocortex is tightly associated with the production of transient amplifying progenitors with greater proliferative capacity by RGPs (Betizeau et al., 2013; Fietz et al., 2010; Hansen et al., 2010; Reillo et al., 2011). It will be interesting to quantitatively determine the proliferative behavior and unit size of RGPs in different species and their relationship to neocortical expansion.

EXPERIMENTAL PROCEDURES

MADM-11^{GT} (JAX Stock 013749) and *MADM-11*^{TG} (JAX Stock 013751) mice were produced as previously described (Hippenmeyer et al., 2010). *Emx1-CreER*^{T2} (Kessaris et al., 2006), *Nestin-CreER*^{T2} (Imayoshi et al., 2006), and *Otx1*$^{+/-}$ (Acampora et al., 1996) mice were kindly provided by Dr. Nicoletta Tekki-Kessaris, Dr. Ryoichiro Kageyama, and Dr. Antonio Simeone, respectively. Mice were bred and maintained according to guidelines established by the institutional animal committees. For MADM labeling, *Emx1-CreER*$^{T2+/-}$*;MADM-11*$^{GT/GT}$ or *Nestin-CreER*$^{T2+/-}$*;MADM-11*$^{GT/GT}$ mice were crossed with *MADM-11*$^{TG/TG}$ mice, and the time of pregnancy was determined by the presence of the vaginal plug (E0). The *Otx1* mutant allele was genetically linked to *MADM-11*^{GT} through meiotic recombination. For clone induction, pregnant females were injected intraperitoneally with TM (Sigma) dissolved in corn oil (Sigma) at E10, E11, E12, and E13 or were orally gavaged with TM at E15 at a dose of 25–50 μg/g of body weight. Live embryos were recovered at E18–E19 through cesarean section, fostered, and raised for further analysis.

Mice were perfused intracardially with 4% paraformaldehyde (PFA) in phosphate-buffered saline (PBS, pH 7.4). Brains were removed and postfixed overnight at 4°C. Serial coronal sections of individual brains were prepared using a vibratome or cryostat (Leica Microsystems) and subjected to immunohistochemistry. The following primary antibodies were used: anti-GFP (Nacalai Tesque), anti-RFP/tdTomato (Rockland), anti-BRN2 (Santa Cruz), anti-CUX1 (Santa Cruz), anti-CTIP2 (Abcam), anti-NURR1 (R&D Systems), and anti-Cleaved Caspase-3 (Promega). Sections were then mounted on glass slides, imaged using confocal microscopy (FV1000, Olympus or LSM700, Zeiss), and reconstructed using Neurolucida, StereoInvetigator (MBF Bioscience), and IMARIS (Bitplane).

For 3D reconstruction, each section was analyzed sequentially in the rostral to caudal order. The boundaries of the entire brain and lateral ventricles were traced and aligned. Individual labeled neurons and glia were represented as colored dots and stars (three to four times the size of the cell body), respectively. Layer boundaries based on nuclear staining were also documented. Cortical areas were identified based on the Allen Brain Atlas (http://mouse.brain-map.org/static/atlas). The NND analysis and the quantitative analysis of clone size and distribution are described in the Extended Experimental Procedures.

Data are presented as mean ± SEM, and statistical differences were determined using nonparametric statistical test (i.e., Mann-Whitney-Wilcoxon and Kruskal-Wallis tests).

SUPPLEMENTAL INFORMATION

Supplemental Information includes Extended Experimental Procedures, seven figures, and four movies and can be found with this article online at http://dx.doi.org/10.1016/j.cell.2014.10.027.

AUTHOR CONTRIBUTIONS

P.G., S.H., L.L., and S.-H.S. conceived the project and designed the experiments. P.G., L.H., R.I., K.C., and O.K. performed *Emx1-CreER*^{T2}*;MADM* experiments, and M.P.P., C.S., E.P., and S.H. performed *Nestin-CreER*^{T2}*;MADM* experiments. T.G.K., C.W., Z.H., K.H., and B.D.S. carried out quantitative analysis of experimental datasets. P.G., T.G.K., B.D.S., L.L., S.H., and S.-H.S. wrote the manuscript with input from all the other authors.

ACKNOWLEDGMENTS

We thank Dr. Nicoletta Tekki-Kessaris, Dr. Ryoichiro Kageyama, and Dr. Antonio Simeone for kindly providing *Emx1-CreER*^{T2}, *Nestin-CreER*^{T2}, and *Otx1*$^{+/-}$ mouse lines, respectively; Dr. Rick Livesey for helpful discussions, Zhizhong Li and Susanne Laukoter for technical support; and Shi and Hippenmeyer lab members for insightful discussions and input. This work was supported by the European Union (FP7-CIG618444 to S.H.), the Simons Foundation (274336 to S.-H.S.), NIH grants (R01DA024681 and R01MH101382 to S.-H.S., R01NS050835 to L.L., and T32HD060600 to L.H.), the Wellcome Trust (098357/Z/12/Z to B.D.S.), and the Human Frontier Science Program (RGP0053 to S.-H.S., S.H., B.D.S., and K.H.). L.L. is an investigator of the Howard Hughes Medical Institute.

Received: June 27, 2014
Revised: September 22, 2014
Accepted: October 10, 2014
Published: November 6, 2014

REFERENCES

Acampora, D., Mazan, S., Avantaggiato, V., Barone, P., Tuorto, F., Lallemand, Y., Brûlet, P., and Simeone, A. (1996). Epilepsy and brain abnormalities in mice lacking the Otx1 gene. Nat. Genet. *14*, 218–222.

Angevine, J.B., Jr., and Sidman, R.L. (1961). Autoradiographic study of cell migration during histogenesis of cerebral cortex in the mouse. Nature *192*, 766–768.

Anthony, T.E., Klein, C., Fishell, G., and Heintz, N. (2004). Radial glia serve as neuronal progenitors in all regions of the central nervous system. Neuron *41*, 881–890.

Bayraktar, O.A., and Doe, C.Q. (2013). Combinatorial temporal patterning in progenitors expands neural diversity. Nature *498*, 449–455.

Betizeau, M., Cortay, V., Patti, D., Pfister, S., Gautier, E., Bellemin-Ménard, A., Afanassieff, M., Huissoud, C., Douglas, R.J., Kennedy, H., and Dehay, C. (2013). Precursor diversity and complexity of lineage relationships in the outer subventricular zone of the primate. Neuron *80*, 442–457.

Brown, K.N., Chen, S., Han, Z., Lu, C.H., Tan, X., Zhang, X.J., Ding, L., Lopez-Cruz, A., Saur, D., Anderson, S.A., et al. (2011). Clonal production and organization of inhibitory interneurons in the neocortex. Science *334*, 480–486.

Clayton, E., Doupé, D.P., Klein, A.M., Winton, D.J., Simons, B.D., and Jones, P.H. (2007). A single type of progenitor cell maintains normal epidermis. Nature *446*, 185–189.

Costa, M.R., Bucholz, O., Schroeder, T., and Götz, M. (2009). Late origin of glia-restricted progenitors in the developing mouse cerebral cortex. Cereb. Cortex *19* (*Suppl 1*), i135–i143.

Desai, A.R., and McConnell, S.K. (2000). Progressive restriction in fate potential by neural progenitors during cerebral cortical development. Development *127*, 2863–2872.

Diggle, P.J. (2003). Statistical Analysis of Spatial Point Patterns (New York: Oxford University Press Inc.).

Dominguez, M.H., Ayoub, A.E., and Rakic, P. (2013). POU-III transcription factors (Brn1, Brn2, and Oct6) influence neurogenesis, molecular identity, and migratory destination of upper-layer cells of the cerebral cortex. Cereb. Cortex *23*, 2632–2643.

Eiraku, M., Watanabe, K., Matsuo-Takasaki, M., Kawada, M., Yonemura, S., Matsumura, M., Wataya, T., Nishiyama, A., Muguruma, K., and Sasai, Y. (2008). Self-organized formation of polarized cortical tissues from ESCs and its active manipulation by extrinsic signals. Cell Stem Cell *3*, 519–532.

Espinosa, J.S., and Luo, L. (2008). Timing neurogenesis and differentiation: insights from quantitative clonal analyses of cerebellar granule cells. J. Neurosci. *28*, 2301–2312.

Fietz, S.A., Kelava, I., Vogt, J., Wilsch-Bräuninger, M., Stenzel, D., Fish, J.L., Corbeil, D., Riehn, A., Distler, W., Nitsch, R., and Huttner, W.B. (2010). OSVZ progenitors of human and ferret neocortex are epithelial-like and expand by integrin signaling. Nat. Neurosci. *13*, 690–699.

Fishell, G., Mason, C.A., and Hatten, M.E. (1993). Dispersion of neural progenitors within the germinal zones of the forebrain. Nature *362*, 636–638.

Florio, M., and Huttner, W.B. (2014). Neural progenitors, neurogenesis and the evolution of the neocortex. Development *141*, 2182–2194.

Franco, S.J., and Müller, U. (2013). Shaping our minds: stem and progenitor cell diversity in the mammalian neocortex. Neuron *77*, 19–34.

Franco, S.J., Gil-Sanz, C., Martinez-Garay, I., Espinosa, A., Harkins-Perry, S.R., Ramos, C., and Müller, U. (2012). Fate-restricted neural progenitors in the mammalian cerebral cortex. Science *337*, 746–749.

Frantz, G.D., and McConnell, S.K. (1996). Restriction of late cerebral cortical progenitors to an upper-layer fate. Neuron *17*, 55–61.

Frantz, G.D., Weimann, J.M., Levin, M.E., and McConnell, S.K. (1994). Otx1 and Otx2 define layers and regions in developing cerebral cortex and cerebellum. J. Neurosci. *14*, 5725–5740.

Gaspard, N., Bouschet, T., Hourez, R., Dimidschstein, J., Naeije, G., van den Ameele, J., Espuny-Camacho, I., Herpoel, A., Passante, L., Schiffmann, S.N., et al. (2008). An intrinsic mechanism of corticogenesis from embryonic stem cells. Nature *455*, 351–357.

Greig, L.C., Woodworth, M.B., Galazo, M.J., Padmanabhan, H., and Macklis, J.D. (2013). Molecular logic of neocortical projection neuron specification, development and diversity. Nat. Rev. Neurosci. *14*, 755–769.

Guo, C., Eckler, M.J., McKenna, W.L., McKinsey, G.L., Rubenstein, J.L., and Chen, B. (2013). Fezf2 expression identifies a multipotent progenitor for neocortical projection neurons, astrocytes, and oligodendrocytes. Neuron *80*, 1167–1174.

Hansen, D.V., Lui, J.H., Parker, P.R., and Kriegstein, A.R. (2010). Neurogenic radial glia in the outer subventricular zone of human neocortex. Nature *464*, 554–561.

He, J., Zhang, G., Almeida, A.D., Cayouette, M., Simons, B.D., and Harris, W.A. (2012). How variable clones build an invariant retina. Neuron *75*, 786–798.

Hippenmeyer, S., Youn, Y.H., Moon, H.M., Miyamichi, K., Zong, H., Wynshaw-Boris, A., and Luo, L. (2010). Genetic mosaic dissection of Lis1 and Ndel1 in neuronal migration. Neuron *68*, 695–709.

Imayoshi, I., Ohtsuka, T., Metzger, D., Chambon, P., and Kageyama, R. (2006). Temporal regulation of Cre recombinase activity in neural stem cells. Genesis *44*, 233–238.

Kessaris, N., Fogarty, M., Iannarelli, P., Grist, M., Wegner, M., and Richardson, W.D. (2006). Competing waves of oligodendrocytes in the forebrain and postnatal elimination of an embryonic lineage. Nat. Neurosci. *9*, 173–179.

Klein, A.M., Nakagawa, T., Ichikawa, R., Yoshida, S., and Simons, B.D. (2010). Mouse germ line stem cells undergo rapid and stochastic turnover. Cell Stem Cell *7*, 214–224.

Kornack, D.R., and Rakic, P. (1995). Radial and horizontal deployment of clonally related cells in the primate neocortex: relationship to distinct mitotic lineages. Neuron *15*, 311–321.

Kriegstein, A., and Alvarez-Buylla, A. (2009). The glial nature of embryonic and adult neural stem cells. Annu. Rev. Neurosci. *32*, 149–184.

Kwan, K.Y., Sestan, N., and Anton, E.S. (2012). Transcriptional co-regulation of neuronal migration and laminar identity in the neocortex. Development *139*, 1535–1546.

Li, X., Erclik, T., Bertet, C., Chen, Z., Voutev, R., Venkatesh, S., Morante, J., Celik, A., and Desplan, C. (2013). Temporal patterning of Drosophila medulla neuroblasts controls neural fates. Nature *498*, 456–462.

Luskin, M.B., Pearlman, A.L., and Sanes, J.R. (1988). Cell lineage in the cerebral cortex of the mouse studied in vivo and in vitro with a recombinant retrovirus. Neuron *1*, 635–647.

Magavi, S., Friedmann, D., Banks, G., Stolfi, A., and Lois, C. (2012). Coincident generation of pyramidal neurons and protoplasmic astrocytes in neocortical columns. J. Neurosci. *32*, 4762–4772.

Marín, O. (2012). Brain development: The neuron family tree remodelled. Nature *490*, 185–186.

McCarthy, M., Turnbull, D.H., Walsh, C.A., and Fishell, G. (2001). Telencephalic neural progenitors appear to be restricted to regional and glial fates before the onset of neurogenesis. J. Neurosci. *21*, 6772–6781.

Noctor, S.C., Martínez-Cerdeño, V., Ivic, L., and Kriegstein, A.R. (2004). Cortical neurons arise in symmetric and asymmetric division zones and migrate through specific phases. Nat. Neurosci. *7*, 136–144.

O'Rourke, N.A., Dailey, M.E., Smith, S.J., and McConnell, S.K. (1992). Diverse migratory pathways in the developing cerebral cortex. Science *258*, 299–302.

Price, J., and Thurlow, L. (1988). Cell lineage in the rat cerebral cortex: a study using retroviral-mediated gene transfer. Development *104*, 473–482.

Qian, X., Goderie, S.K., Shen, Q., Stern, J.H., and Temple, S. (1998). Intrinsic programs of patterned cell lineages in isolated vertebrate CNS ventricular zone cells. Development *125*, 3143–3152.

Qian, X., Shen, Q., Goderie, S.K., He, W., Capela, A., Davis, A.A., and Temple, S. (2000). Timing of CNS cell generation: a programmed sequence of neuron and glial cell production from isolated murine cortical stem cells. Neuron *28*, 69–80.

Rakic, P. (1988). Specification of cerebral cortical areas. Science *241*, 170–176.

Reillo, I., de Juan Romero, C., García-Cabezas, M.A., and Borrell, V. (2011). A role for intermediate radial glia in the tangential expansion of the mammalian cerebral cortex. Cereb. Cortex *21*, 1674–1694.

Rowitch, D.H., and Kriegstein, A.R. (2010). Developmental genetics of vertebrate glial-cell specification. Nature *468*, 214–222.

Schmechel, D.E., and Rakic, P. (1979). A Golgi study of radial glial cells in developing monkey telencephalon: morphogenesis and transformation into astrocytes. Anat. Embryol. (Berl.) *156*, 115–152.

Shen, Q., Wang, Y., Dimos, J.T., Fasano, C.A., Phoenix, T.N., Lemischka, I.R., Ivanova, N.B., Stifani, S., Morrisey, E.E., and Temple, S. (2006). The timing of cortical neurogenesis is encoded within lineages of individual progenitor cells. Nat. Neurosci. *9*, 743–751.

Snippert, H.J., van der Flier, L.G., Sato, T., van Es, J.H., van den Born, M., Kroon-Veenboer, C., Barker, N., Klein, A.M., van Rheenen, J., Simons, B.D., and Clevers, H. (2010). Intestinal crypt homeostasis results from neutral competition between symmetrically dividing Lgr5 stem cells. Cell *143*, 134–144.

Tan, S.S., Kalloniatis, M., Sturm, K., Tam, P.P., Reese, B.E., and Faulkner-Jones, B. (1998). Separate progenitors for radial and tangential cell dispersion during development of the cerebral neocortex. Neuron *21*, 295–304.

Teixeira, V.H., Nadarajan, P., Graham, T.A., Pipinikas, C.P., Brown, J.M., Falzon, M., Nye, E., Poulsom, R., Lawrence, D., Wright, N.A., et al. (2013). Stochastic homeostasis in human airway epithelium is achieved by neutral competition of basal cell progenitors. eLife *2*, e00966.

Walsh, C., and Cepko, C.L. (1988). Clonally related cortical cells show several migration patterns. Science *241*, 1342–1345.

Walsh, C., and Cepko, C.L. (1992). Widespread dispersion of neuronal clones across functional regions of the cerebral cortex. Science *255*, 434–440.

Weimann, J.M., Zhang, Y.A., Levin, M.E., Devine, W.P., Brûlet, P., and McConnell, S.K. (1999). Cortical neurons require Otx1 for the refinement of exuberant axonal projections to subcortical targets. Neuron *24*, 819–831.

Zong, H., Espinosa, J.S., Su, H.H., Muzumdar, M.D., and Luo, L. (2005). Mosaic analysis with double markers in mice. Cell *121*, 479–492.

Supplemental Information

EXTENDED EXPERIMENTAL PROCEDURES

The Nearest Neighbor Distance Analysis

The distribution of the nearest neighbor distance (NND) reflects the spatial point pattern of the data set, as previously described (Diggle, 2003). Specifically, given *N* cells in a data set, for each cell *i* the distance to its closest neighbor was measured and denoted as $\boldsymbol{d_i}$, the NND for cell ***i***. The indicator function *f(y,d)* was then calculated as:

$$f(y,d)=\begin{cases}1, & \text{if } d\leq y,\\ 0, & \text{otherwise}.\end{cases}$$

Thus, the cumulative distribution function (CDF) of NND is:

$$G(y)=\sum_{i=1}^{N} f(y,d_i).$$

In this analysis, the shorter NND distance reflects clustering, whereas the longer NND distance reflects dispersing. Simulated random data sets contained the same number of data points within the same volume (i.e., the neocortex) as the experimental data sets, and were repeated 100 times.

Quantitative Analysis of Clone Size and Distribution

Fitting of the Gaussian to the asymmetric neurogenic clone size distribution was performed by least-squares using MATLAB (MathWorks). The fitting error was calculated as the ratio of the residual area to the area under the fitting curve, after smoothing the data with window size 3. For the total distribution of clones labeled at E10-12, the sum of a series of progressive convolutions of the Unitary Gaussian was fitted to clones up to a size of 50 neurons (192 out of 235 clones). To take into account limited clone numbers, 95% plausible intervals for the predicted frequency *D(n)* of clones of size *n* were estimated as

$$D(n)\pm 2\sqrt{D(n)\times(1-D(n))/N},$$

where *N* denotes the total number of clones.

The frequency distribution of proliferative clone sizes was approximated as

$$p(m)=\frac{1}{\langle m\rangle}e^{-\frac{m}{\langle m\rangle}},$$

where *m* denotes the size of the clone and $\langle m\rangle$ denotes the average total clone size at the given time point. Based on the correlation between sister subclone sizes, RGP behavior is approximately synchronized within one clone, so the number of rounds of proliferative division, *n*, of a clone of size *m* from the time of labeling was estimated based on the relationship

$$m=2^n\times\mu,$$

where μ denotes the mean of the unitary neuronal output. The distribution of *n*, denoted by *P(n),* followed as

$$P(n)=P\left(\log_2\frac{m}{\mu}\right).$$

P(n) was smoothened by a moving average of width one division. To take into account that it is relatively more likely to label RGP lineages that have proliferated more, the distribution of proliferative capacity of the founder RGPs was adjusted as

$$F(n)=\ln(2)\cdot\left(\frac{2^n}{\langle 2^n\rangle}\right)^2 e^{-\frac{2^n}{\langle 2^n\rangle}}.$$

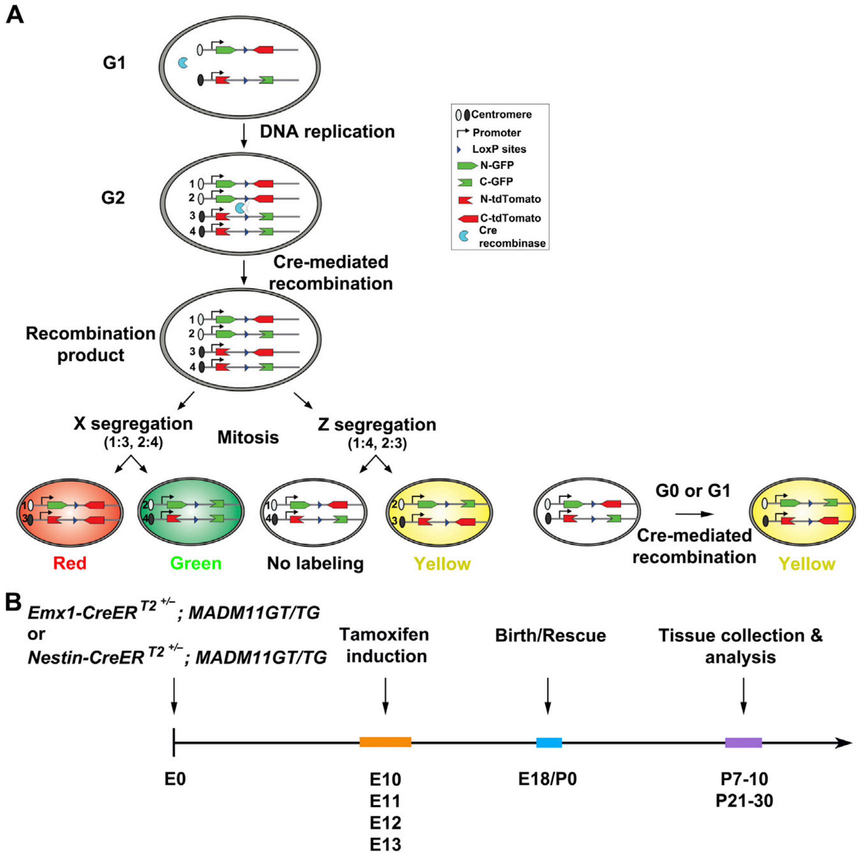

Figure S1. Outline of MADM-Based Clonal Analysis of Neocortical Excitatory Neuron Production and Organization, Related to Figure 1

(A) Schematic of MADM labeling.

(B) Experimental paradigm of MADM-based clonal analysis. A single dose of TM treatment is performed at E10, E11, E12 or E13, and brains are analyzed at P7-10, when neuronal migration in the neocortex is mostly finished, or P21-30, when neocortical development is largely complete.

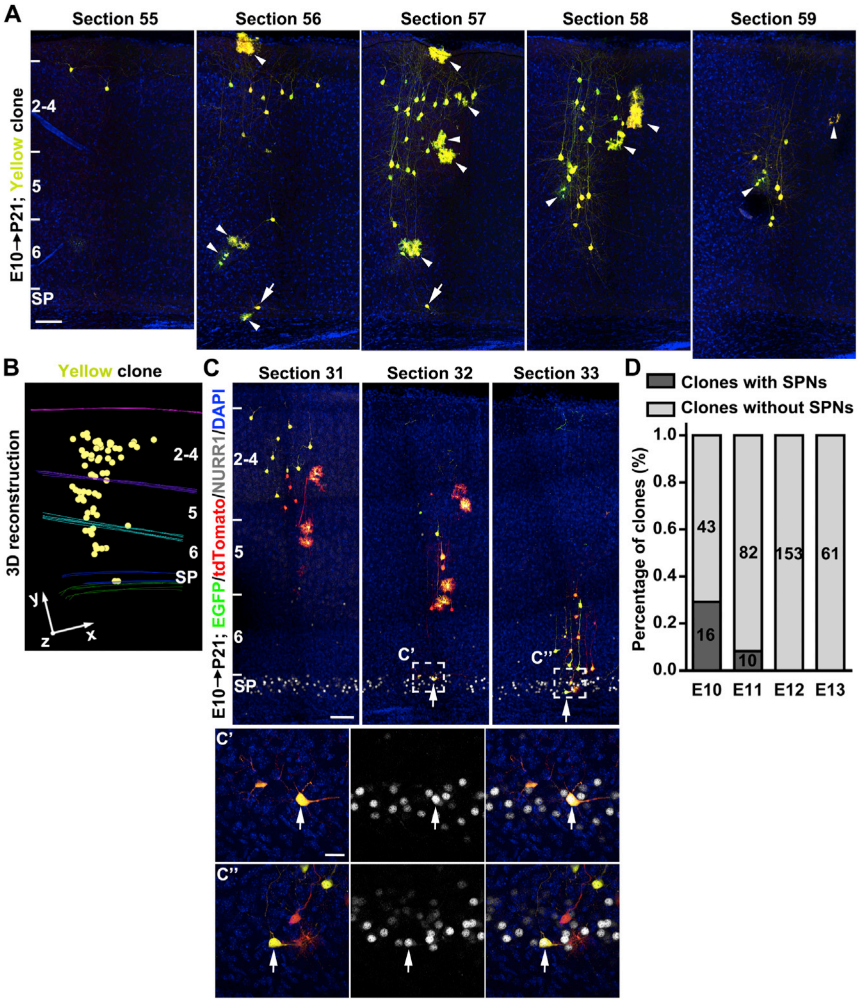

Figure S2. MADM Labeling of Yellow Clones and Clones with Subplate Neurons in the Neocortex, Related to Figure 1

(A) Confocal images of a yellow clone in Figure 1A. Arrowheads indicate glial cells and arrows indicate two neurons in the subplate zone. Scale bar: 100 μm.

(B) 3-D reconstruction image of neurons in the yellow clone.

(C) Confocal images of a yellow clone containing two subplate neurons (SPNs, arrows) expressing NURR1 (white). High magnification images of SPNs (broken lines, C' and C'') are shown at the bottom. Scale bars: 100 μm and 25 μm.

(D) Percentage of clones labeled at different embryonic stages containing SPNs.

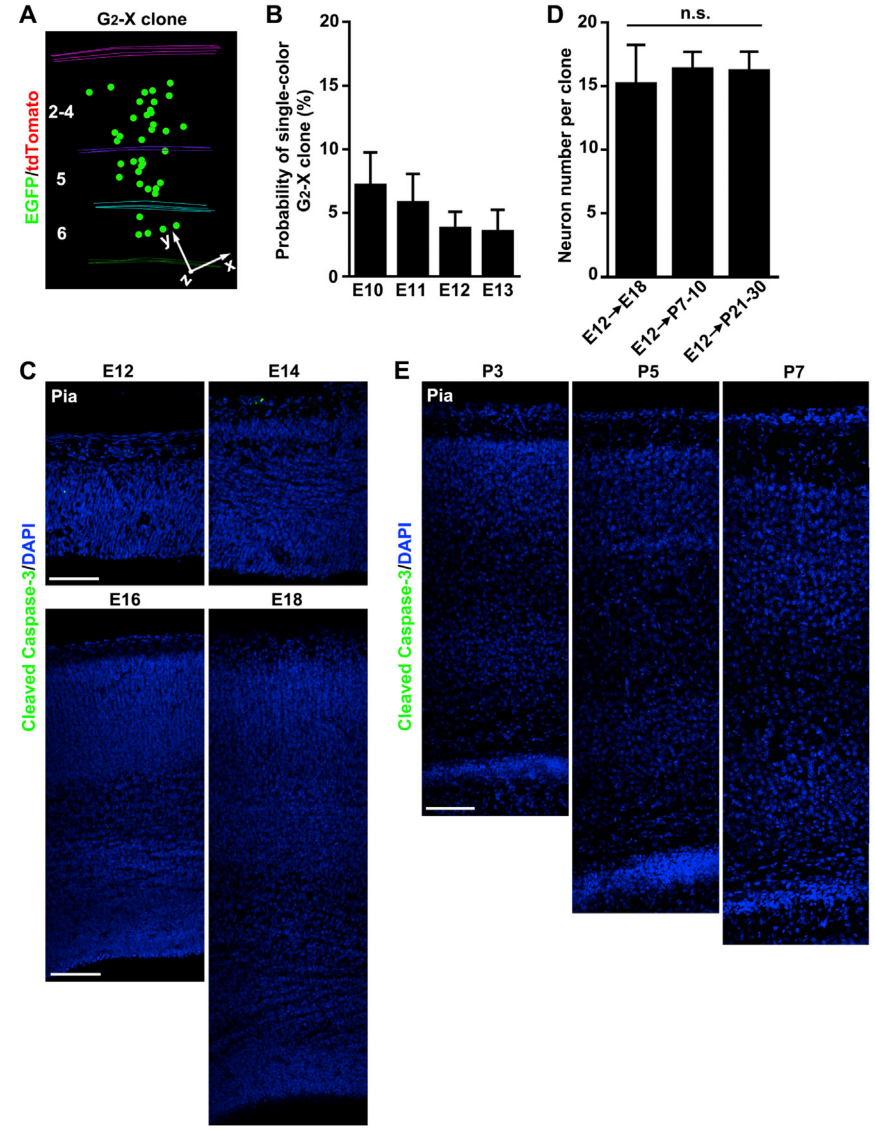

Figure S3. No Substantial Apoptosis in the Embryonic and Postnatal Neocortex, Related to Figure 2

(A) 3-D reconstruction image of a single colored (green fluorescent only) G_2-X clone.

(B) Quantification of apoptosis rate of the daughter cell of dividing progenitors inferred from the occurrence of single colored G_2-X clones. Data are presented as mean ± SEM. Note that the rate of the daughter cell apoptosis is less than 4%–7%.

(C) Confocal images of E12, E14, E16 and E18 neocortices stained with the antibody against Cleaved Caspase-3 (green), a marker for apoptotic cells, and with DAPI (blue). Note no substantial apoptosis in the embryonic neocortex. Scale bar: 100 μm.

(D) Quantification of the size of clones labeled at E12 and analyzed at E18, P7-10 and P21-30. Data are presented as mean ± SEM. n.s., not significant. Note that the clonal size is similar at different postnatal stages, indicating no substantial excitatory neuron apoptosis postnatally.

(E) Confocal images of P3, P5 and P7 neocortices stained with the antibody against Cleaved Caspase-3 (green) and with DAPI (blue). Scale bar: 100 μm.

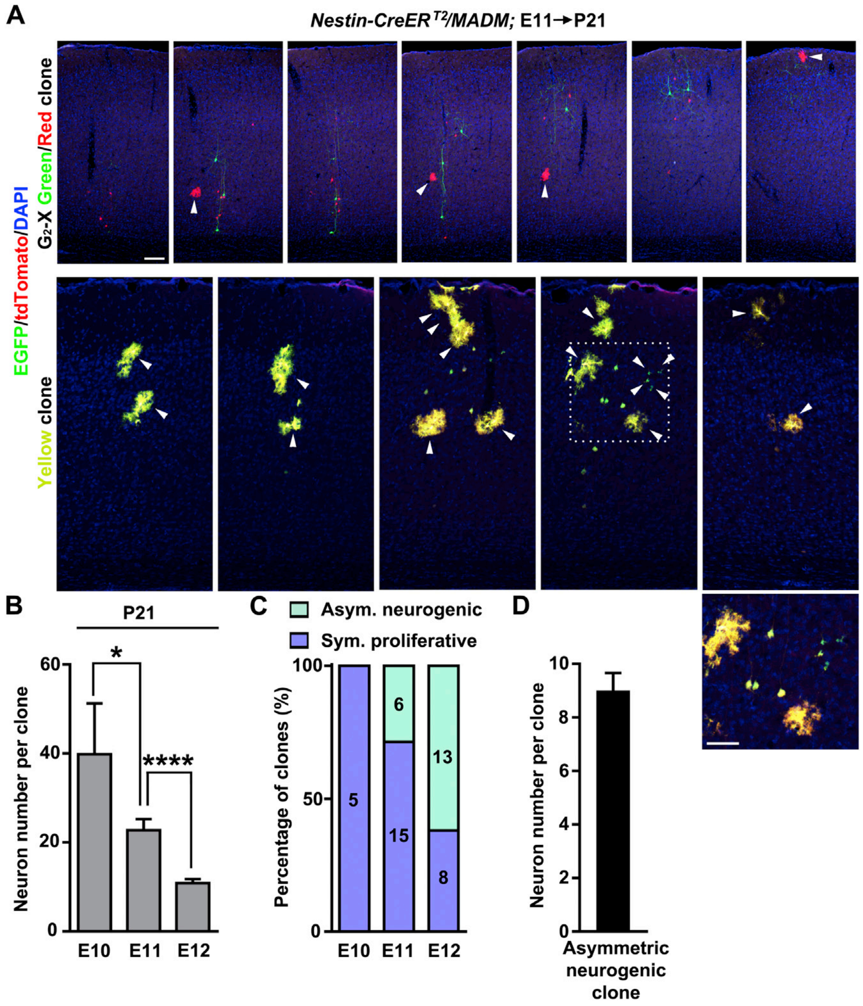

Figure S4. Neocortical Excitatory Neuron Clones Labeled Using *Nestin-CreER^{T2}/MADM*, Related to Figure 2

(A) Confocal images of a green/red G_2-X clone (top) and a yellow clone (bottom) in a P21 brain treated with TM at E11. Arrowheads indicate glial cells. A high magnification image of labeled neurons and glia (broken lines) is shown as an inset. Scale bars: 100 μm.

(B) Quantification of the clone size (E10, n = 5; E11, n = 21; E12, n = 21). Data are presented as mean ± SEM. (*$p < 0.05$; ****$p < 0.0001$).

(C) Percentage of symmetric proliferative division versus asymmetric neurogenic division at different embryonic stages.

(D) Quantification of the size of asymmetric neurogenic clones (n = 19). Data are presented as mean ± SEM.

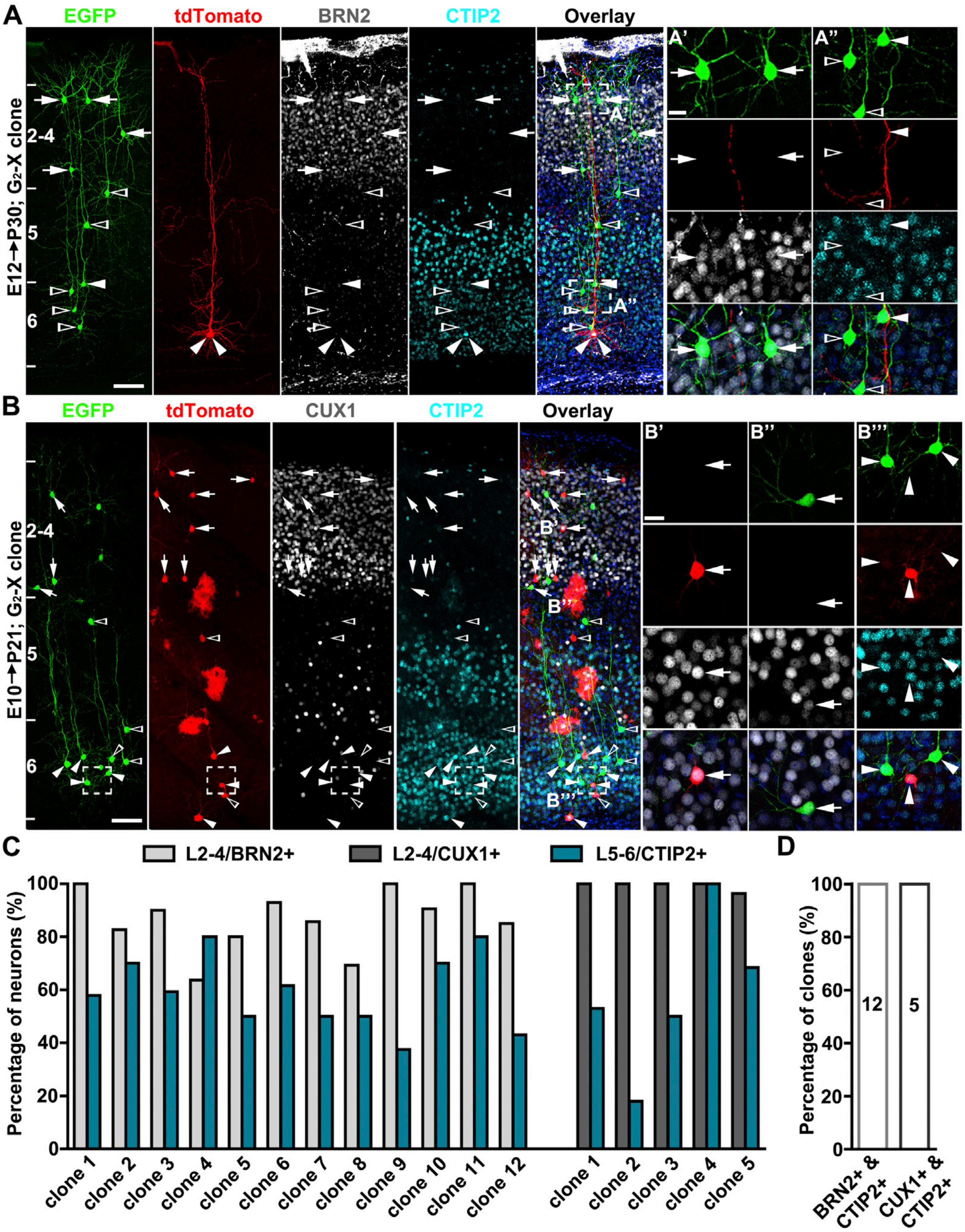

Figure S5. G_2-X Clones Contain Both Superficial- and Deep-Layer Neurons, Related to Figure 4

(A) Confocal images of a representative asymmetric neurogenic G_2-X clone stained with the antibodies against EGFP (green), tdTomato (red), BRN2 (white) and CTIP2 (cyan), and with DAPI (blue). High magnification image of representative superficial (A') and deep (A'') layer neurons (broken lines) are shown to the right. Arrows indicate neurons positive for BRN2, arrowheads indicate neurons positive for CTIP2, and open arrowheads indicate neurons negative for BRN2 or CTIP2. Scale bars: 100 μm and 20 μm.

(B) Confocal images of a representative symmetric proliferative G_2-X clone stained with the antibodies against EGFP (green), tdTomato (red), CUX1 (white) and CTIP2 (cyan), and with DAPI (blue). Arrows indicate neurons positive for CUX1, arrowheads indicate neurons positive for CTIP2, and open arrowheads indicate neurons negative for CUX1 or CTIP2. High magnification images of representative superficial (B' and B'') and deep (B''', broken lines) layer neurons are shown to the right. Scale bars: 100 μm and 20 μm.

(C) Percentage of L2-4 and L5-6 neurons of individual clones labeled at E10-12 that are positive for BRN2+ or CUX1+ and CTIP2+, respectively. Note all the clones contain BRN2+ or CUX1+ and CTIP2+ neurons.

(D) Percentage of clones labeled at E10-12 that contain both BRN2+ or CUX1+ and CTIP2+ neurons.

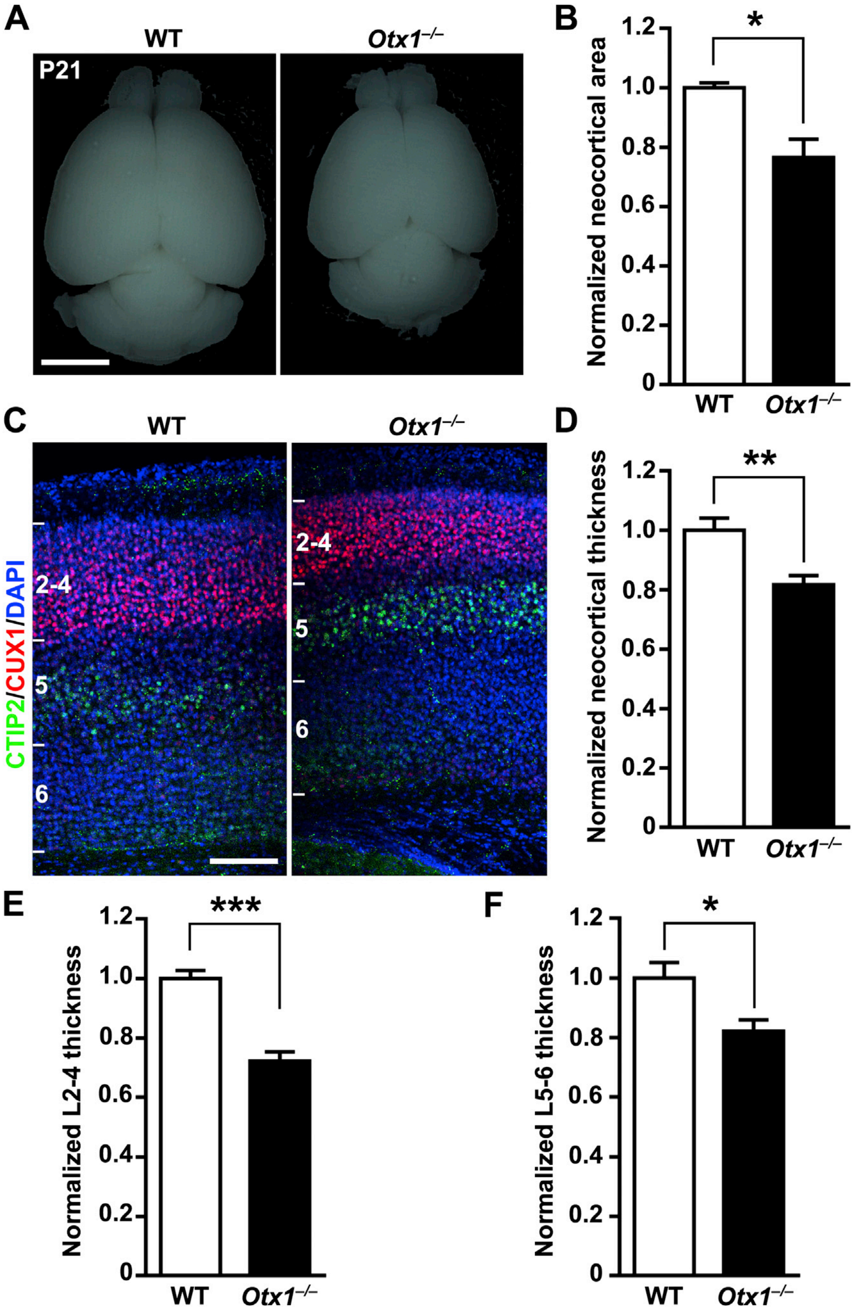

Figure S6. Microcephaly and Reduction in Neocortical Thickness in *Otx1*$^{-/-}$ Mice, Related to Figure 5

(A) Whole-mount images of representative P21 wild-type (*Otx1*$^{+/+}$) and *Otx1*$^{-/-}$ brains. Note the clear microcephaly of *Otx1*$^{-/-}$ brains. Scale bar: 500 μm.

(B) Quantification of the area of the neocortex. Note ~25% reduction in the neocortical area of *Otx1*$^{-/-}$ brains compared to wild-type littermate controls. Data are presented as mean ± SEM. (n = 4; *p < 0.05).

(C) Confocal images of representative P11 wild-type and *Otx1*$^{-/-}$ neocortices stained for CUX1 (red) and CTIP2 (green), the superficial and deep layer neuron markers, and with DAPI (blue). Note the reduction in the overall thickness of the neocortex as well as the thickness of both the superficial (2-4) and deep (5-6) layers. Scale bar: 100 μm.

(D–F) Quantification of the thickness of all layers (D), and the superficial (E) and deep (F) layers across different neocortical areas. Data are presented as mean ± SEM. (3 regions along the dorsolateral axis from 4 sections were analyzed for each condition; *p < 0.05; **p < 0.01; ***p < 0.001).

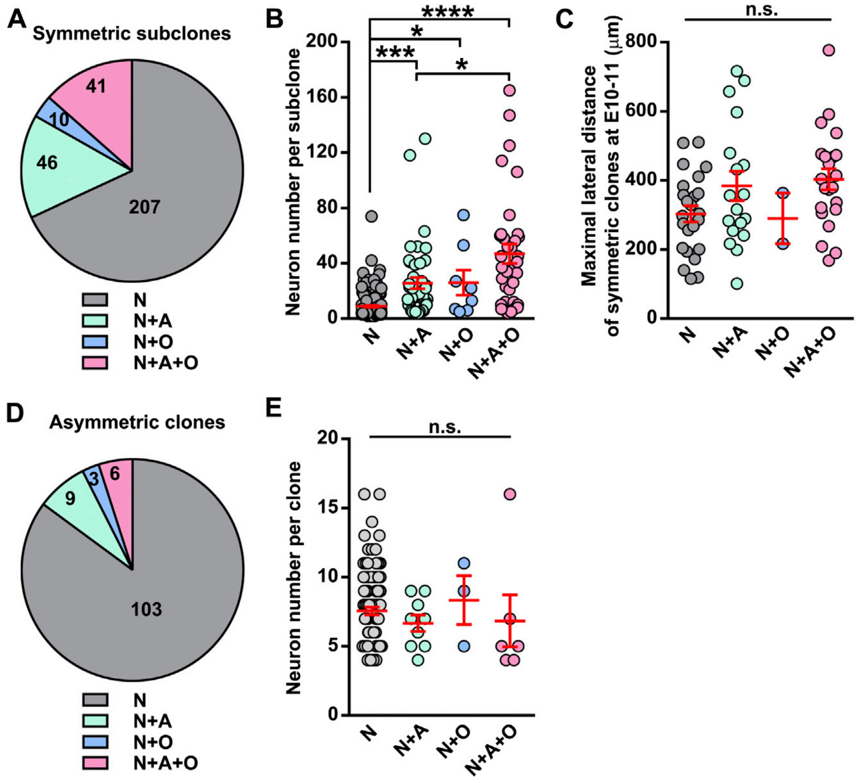

Figure S7. Relationship between Neurogenesis and Gliogenesis, Related to Figure 7

(A) Percentage of symmetric subclones that contain neurons only (N), neurons and astrocytes (N+A), neurons and oligodendrocytes (N+O) or neuron, astrocytes and oligodendrocytes (N+A+O).

(B) Number of neurons per subclones that contain N, N+A, N+O or N+A+O. Each cycle represents one subclone and red lines represent mean ± SEM. ****$p < 0.0001$; ***$p < 0.001$; *$p < 0.05$.

(C) Maximal lateral distance between neurons of symmetric subclones that contain N, N+A, N+O or N+A+O. n.s., not significant.

(D) Percentage of asymmetric neurogenic clones that contain N, N+A, N+O or N+A+O.

(E) Number of neurons per asymmetric neurogenic clones that contain N, N+A, N+O or N+A+O. n.s., not significant.
